# Current progress of cerebral organoids for modeling Alzheimer's disease origins and mechanisms

**DOI:** 10.1002/btm2.10378

**Published:** 2022-08-02

**Authors:** Sai Sreenivasamurthy, Mahek Laul, Nan Zhao, Tiffany Kim, Donghui Zhu

**Affiliations:** ^1^ Department of Biomedical Engineering Stony Brook University Stony Brook New York USA; ^2^ Institute for Nanobiotechnology Johns Hopkins University Baltimore Maryland USA

**Keywords:** amyloid beta, familial Alzheimer's, neurodegeneration, neurofibrillary tangles, neurospheroids, sporadic Alzheimer's

## Abstract

Alzheimer's disease (AD) is a progressive, neurodegenerative disease that has emerged as a leading risk factor for dementia associated with increasing age. Two‐dimensional (2D) cell culture and animal models, which have been used to analyze AD pathology and search for effective treatments for decades, have significantly contributed to our understanding of the mechanism of AD. Despite their successes, 2D and animal models can only capture a fraction of AD mechanisms due to their inability to recapitulate human brain‐specific tissue structure, function, and cellular diversity. Recently, the emergence of three‐dimensional (3D) cerebral organoids using tissue engineering and induced pluripotent stem cell technology has paved the way to develop models that resemble features of human brain tissue more accurately in comparison to prior models. In this review, we focus on summarizing key research strategies for engineering in vitro 3D human brain‐specific models, major discoveries from using AD cerebral organoids, and its future perspectives.

## 1 INTRODUCTION

Alzheimer's disease (AD) is a cognitive disorder that affects approximately 5.8 million Americans. It is characterized by memory loss, confusion, lack of awareness, and other symptoms that disrupt daily life and lead to a loss of independence.^1^ AD mainly affects the elderly population with approximately 80% of cases associated with those 75 or older.^2^ Unfortunately, there is no cure for AD, and the cost of care associated with AD patients can put an economic strain on caregivers. For example, one study reports average, monthly expenses are €2450 using the proxy good method and €3102 with the opportunity cost method, but averages vary based on the severity of AD.^3^

AD can be classified into two main factions: Familial AD (FAD) and Sporadic AD (SAD). SAD comprises most AD cases, however, FAD is an early‐onset subtype (symptoms seen in patients as early as their 40s or 50s) and affects less than 1% of the overall AD population.^4^ Despite their varying genetic origins, FAD and SAD produce similar pathologies including amyloid beta (Aβ) plaques (specifically a higher ratio of Aβ42 to Aβ40), hyperphosphorylation of the microtubule‐associated protein, tau (P‐tau), aggregation of P‐tau into neurofibrillary tangles (NFTs), abnormal endosomes, increased reactive oxygen species (ROS), neuronal inflammation, and apoptosis.^5^

**FIGURE 1:**
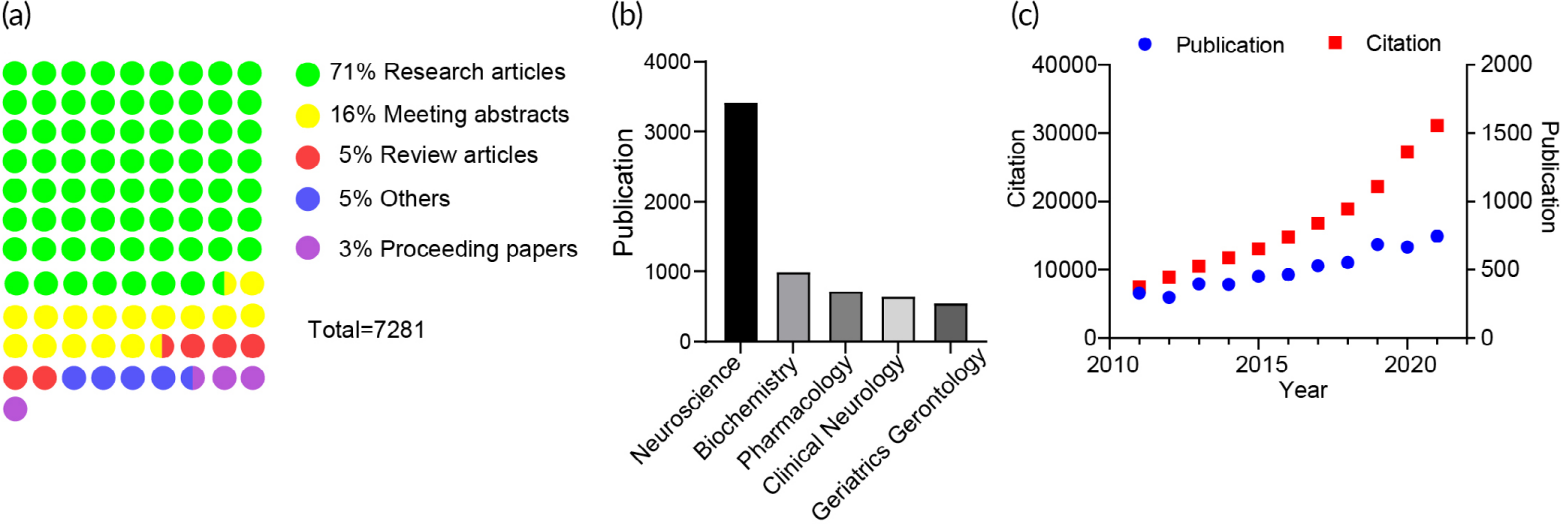
Publication trends for modeling Alzheimer's disease over the past 12 years. (a) Dot‐plot of publication distribution based on types of documents. (b) Summary of publication distribution based on Web of Science Categories. (c) Number of publications versus citations over the past 12 years. Literature search was done on Web of Science using “Alzheimer's disease” and “model” on January 23, 2022.

Significant progress has been made using various cell culture and animal models to understand the pathways behind FAD and SAD progression. Over the past 12 years, 7281 papers related to modeling AD have been published across a range of journal categories (Web of Science; Figure 1a,b). Both the publication number and citation number increased yearly (Figure 1c), indicating a rise in popularity of designing more accurate AD brain models as reliable platforms. 2D and 3D culture models have demonstrated Aβ plaque and NFT formation,^6^, ^7^, ^8^, ^9^, ^10^, ^11^, ^12^, ^13^ however, the structural complexity of the cellular networks is lacking, making results difficult to fully translate in vivo. Animal models have been more successful in modeling AD due to their high physiological relevance,^14^ but these models are not able to fully recapitulate the structure, function, and cellular diversity of human brains.

From advances in tissue engineering and stem cell technology, the development of stem cell‐derived cerebral organoids has provided new strategies to accurately and efficiently model AD pathways in vitro by invoking similar pathologies observed in AD patients. Organoids are self‐assembled 3D structures that mimic in vivo characteristics of a chosen organ; their emergence provides more sophisticated methods to examine tissue structure, pathology, and drug delivery mechanisms. AD organoid models can accurately replicate disease characteristics seen in AD patients and be tailored to mimic FAD, SAD, or alternative AD mechanisms based on risk‐associated genes in the initial cell sources or through manipulation of the microenvironment as the organoids mature.^15^ Additionally, organoid platforms may reduce the reliance on animal models and associated costs, therefore proving to be more ethical and economical.^16^ This short review discusses the current advancements in FAD and SAD organoid models along with future perspectives in this field.

## 2 AD PATHOLOGY

**FIGURE 2:**
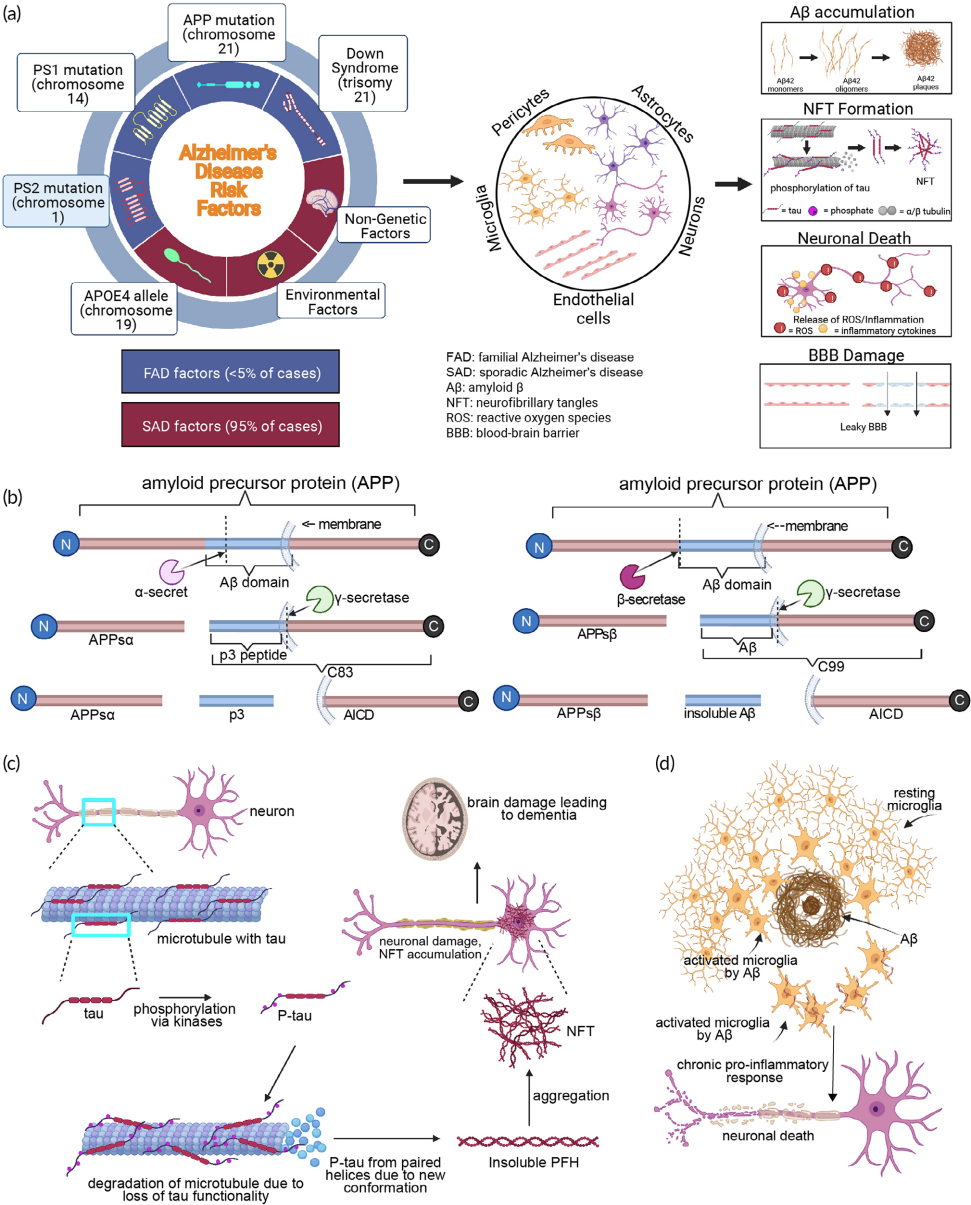
Risk factors of Alzheimer's disease (AD) and associated pathology at cellular and molecular levels. (a) Schematic summary of AD‐associated factors. Genetic, nongenetic, and environmental factors contribute to the development of AD and can affect the functions of varying types of brain cells. Hallmark pathologies for AD include Aβ accumulation, formation of NFTs, BBB damage, and neuronal death. (b) Amyloidogenic pathway (left) and non‐amyloidogenic pathway (right) of amyloid precursor protein (APP) breakdown. (c) Pathology of NFT aggregation. In normal pathology, tau proteins remain bound to microtubules assisting in structural integrity and cell‐to‐cell communication. In AD pathology, tau proteins become hyperphosphorylated and eventually aggregate to form NFTs in neurons. (d) Activated microglial response. With Aβ plaque accumulation, microglia become triggered to release pro‐inflammatory cytokines which compromise neuronal viability. Created with BioRender.com

Many factors could contribute to the development of AD such as genetic factors, environmental toxins, infections, and aging (Figure 2a). FAD patients may begin to exhibit symptoms between their 40s to mid‐50s.^17^ In the case of SAD, the first symptoms appear in the mid‐60s.^18^ Once symptoms show, the progression follows the typical trends associated with AD, though there is variability in which symptoms appear first. In most cases, issues relating to memory and mild cognitive impairment (MCI) are seen in the early stages of AD. MCI includes difficulty with thinking and judgment such as losing train of thought, losing items, impulsive decision‐making, and forgetting important events or appointments. However, the severity of these symptoms is lower and they will not interfere with daily life and personal relationships.^19^ As a patient progresses from mild to moderate AD, memory issues become more severe, leading to repetitive statements/questions, inability to recognize loved ones, difficulty in learning new information/tasks, or inability to create new memories. The patient may also become unaware of their surroundings and therefore, become paranoid or delusional. In the final stages of AD, there is a loss of motor control, affecting the patient's ability to communicate, eat, and control their bowels and bladder. Other symptoms may include seizures, increased sleep, and incoherent noises such as groaning and grunting. The exact timeline for disease progression varies from patient to patient. However, most patients do not survive for longer than 4–8 years after being diagnosed clinically, with diagnosis only being confirmed with postmortem neuropathological analysis.^20^

The hallmark pathologies of AD include the formation and accumulation of extracellular Aβ plaques around neurons, P‐tau, and aggregation of intracellular P‐tau into neurofibrillary tangles (NFT; Figure 2a). Accumulation of Aβ plaques and NFTs may affect neural viability and compromise the effectivity of the blood–brain barrier (BBB).^21^ The BBB is highly selective and is only permeable to certain small lipophilic molecules, oxygen, and carbon dioxide. Aβ plaque formation disrupts surrounding endothelial cells as well as cells adjacent to the BBB such as pericytes, smooth muscle cells, and glia, yielding a “leakier” BBB to blood cells and an overall decline in BBB functionality.^21^, ^22^, ^23^ The neuroinflammation and reactive gliosis caused by the accumulation of Aβ plaques also lead to neuronal and synaptic dysfunction as a result of the inflammatory cytokines released by microglia and astrocytes, producing the cognitive defects seen in AD.^24^

The genes affecting FAD and SAD vary but result in similar pathologies that characterize AD. The gene coding for APP is often mutated in FAD patients, along with PSEN1 and PSEN2 (Table 1). One gene that is commonly found to influence SAD is APOE which codes for apolipoprotein E. The ε3 allele of APOE is the version typically carried, however, the presence of the ε4 allele poses as a risk factor for AD as it is associated with increased Aβ plaque formation. Other genes are also thought to be responsible for influencing SAD development (Table 2).^25^

**TABLE 1:** FAD‐associated genes and functions

APP	Codes for amyloid precursor protein, found abundantly in brain and CNS
PSEN1	Codes for presenilin 1, part of the gamma‐secretase enzyme complex, regulates downstream processes by processing regulatory proteins
PSEN2	Codes for presenilin 2, part of the gamma‐secretase enzyme complex, thought to have a role in transmitting signals from cell membrane to nucleus

**TABLE 2:** SAD‐associated genes and functions

APOE	Codes for apolipoprotein e; e4 allele of this gene increase the risk of developing AD; the most commonly associated gene with SAD
ABCA7	Codes for ATP‐binding cassette A7, plays a role in transmembrane transport activity; linked to AD but the role is unclear
CLU	Codes for apolipoprotein J; responsible for clearance of debris, for example, amyloid beta plaques
CR1	Codes for complement C3b/C4b receptor 1, mediates immune response
PICALM	Codes for phosphatidylinositol binding clathrin assembly protein; important for interneuron communication
PLD3	Codes for phospholipase D3, influences APP processing
TREM2	Triggering receptor, regulates brain's inflammation response
SORL1	Codes for a sorting receptor involved with APP, some variations on chromosome 11 of this gene correlate with AD

Amyloid precursor protein (APP) is a transmembrane protein whose role is not completely understood. Under normal circumstances, APP is broken down by the nonamyloidogenic path in which α‐secretase will locate the Aβ domain on the protein and cleave it between the 16th and 17th amino acid. This produces a soluble neuroprotective N‐terminal fragment called soluble amyloid precursor protein (APPsα).^26^, ^27^ The C‐terminal fragment (C83), which is still attached to the membrane, will be further cleaved by γ‐secretase. These two cuts result in a p3 peptide and intracellular domain of APP (AICD) which does not pose a threat to brain tissues (Figure 2b).^27^ In AD patients, β‐secretase is the primary cleaver of the APP protein. β‐Secretase locates the Aβ domain and cleaves it at the N‐terminus; the cleaved part of the molecule is APPsβ, and the fragment remaining in the membrane is C99.^27^, ^28^ γ‐Secretase functions as normal and cleaves the C‐terminus side of the domain, resulting in the entire Aβ domain dislodging as one insoluble piece, most commonly as isoforms Aβ40 and Aβ42. Aβ40 is generally benign, but Aβ42 is highly self‐aggregating.^27^, ^29^ This mechanism comprises the amyloidogenic pathway which is seen at much higher rates in AD patients. This is evidenced by the accumulation of the insoluble Aβ peptides around neurons in AD patients (Figure 2b). Although the exact mechanism of increased β‐secretase activity in AD patients is unknown, it is known that the Swedish mutation of substituting methionine for leucine at the P1 position of APP enhances β‐secretase cleavage.^30^ It is speculated that the products of the amyloidogenic pathway may have cell signaling mechanisms that also enhance β‐secretase activity. α‐Secretase expression does not decrease, rather there is increased competition between the two enzymes, and the APP mutation provides an advantage to β‐secretase.^31^

The tau protein has many sites with phosphorylation potential. In a healthy physiological environment, the tau proteins are phosphorylated and dephosphorylated by kinases and phosphatases, respectively (Figure 2c). In AD brain tissue, combinations of kinases, cdk5/GSK3, and calcium calmodulin kinase II are largely responsible for the increased rate of proline‐directed phosphorylation. Proteins responsible for dephosphorylation such as phosphatase 2A are unable to balance the phosphorylation. Thus, the tau proteins become hyperphosphorylated and change shape to form paired helical filaments (PHFs).^32^, ^33^ Aggregation of PHFs leads to the development of insoluble NFTs. Tau proteins also lose their functionality to bind cytoskeletal microtubules.^34^ The mechanism by which tau proteins become susceptible to hyperphosphorylation is yet to be confirmed, however, one proposed mechanism is that calpain cleaves tau at the N‐terminus, changing the conformation of the protein from unfolded to a β‐sheet. This conformational change leaves the C‐terminus vulnerable to cleavage by caspase. Once cleaved, tau becomes more susceptible to hyperphosphorylation (Figure 2c).^33^ Additionally, studies have shown a correlation between Aβ plaques and NFT formation, suggesting that there may be a synergistic effect involving these two pathologies.^35^ The buildup of Aβ plaques and NFTs may eventually compromise neuronal viability by microglial activation and high production of pro‐inflammatory cytokines. While early stimulation of microglia provides a more defensive mechanism to remove Aβ debris, a continuous buildup may shift microglia to take a more pro‐inflammatory stance, leading to downstream neuroinflammation and apoptosis (Figure 2d).^36^, ^37^

## 3 MODEL SYSTEMS FOR AD

**FIGURE 3:**
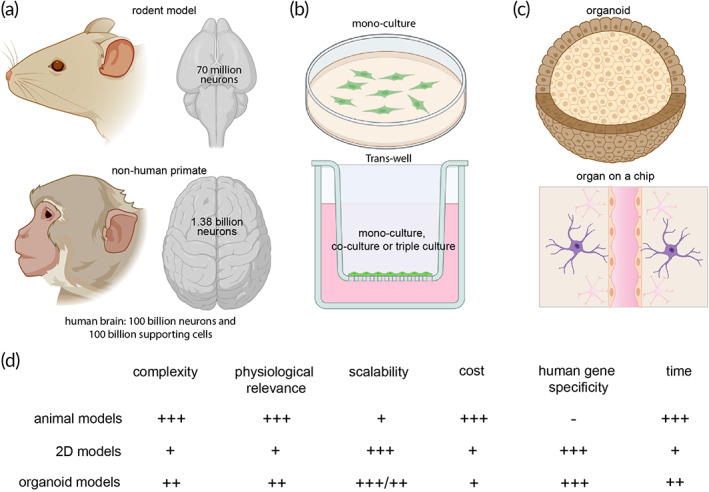
Model systems for Alzheimer's disease. (a) Animal models. Genetically engineered animal models that express AD‐related genes. Two most popular animal models for AD are rodents and nonhuman primates. (b) 2D model systems. Mono‐culture on a petri dish, co‐culture, or tri‐culture of brain cells or a trans‐well insert using primary cells from human patients or induced pluripotent stem cells (iPSCs) derived brain cells. (c) Brain organoid models and brain organ on a chip model. (d) Comparison of animal models, 2D models, and organoids models. −, none; +, low; ++, medium; +++, high

Common model systems that have been used for assessing the mechanism of AD include animal models, 2D cell culture models, 3D organoid models, as well as computational simulations (in silico model). Figure 3 summarizes some key examples of these model systems.

### 3.1 Animal models

AD has been modeled in several animals including fruit flies (*Drosophila*), nematodes (*Caenorhabditis elegans*), zebrafish (*Danio rerio*), primates, and most notably, mice. Modeling with invertebrates can be relevant to human physiology by studying the molecular pathways underlying AD. There is also an advantage in efficiency as invertebrates tend to reproduce much faster than mammals. Fruit flies, nematodes, and zebrafish all have genes that are either homologs or orthologs of some of the genes relevant to AD found in humans (Table 3).^38^, ^39^, ^40^ Although invertebrate models may be useful for learning about disease pathways, they are not ideal for studying treatments as the results would not immediately translate to human applications.

**TABLE 3:** Genes relevant to AD across observed species

Human/primate/mouse	Fruit fly	Nematode	Zebrafish
APP	dAPPL	apl‐1	appa/appb
PSEN1/PSEN2	dPsn	sel‐12. hop‐1	psen1/psen2
MAPT	dTau	ptl‐1	mapta/maptb

Transgenic mice have been used to model and study AD for over two decades.^41^, ^42^, ^43^, ^44^, ^45^, ^46^, ^47^, ^48^ Studying FAD is commonly achieved from cDNA‐based or genome‐based transgenic approaches. Games et al. produced a mouse model, termed PD‐APP, with the APP V717I mutation using the cDNA approach.^43^ Further studies using transgenic mice by Irizarry et al. showed significant Aβ plaque formation, especially in the cortical region. This is consistent with the pathology of AD in humans, but neither NFTs nor neuronal loss was observed.^45^ Interestingly, the mice did display behavioral impairment in tasks relating to spatial memory correlated with Aβ plaque formation as shown by Chen and colleagues using a water‐maze experiment.^42^ Another model created by Novartis Pharmaceuticals also used cDNA‐based transgenics but with K670N/M671L and V717I mutations; this model is termed APP 22 and demonstrated neuronal cell loss around the Aβ plaques, particularly in the highly cellularly dense area of the hippocampus. NFTs were also not observed in this model.^48^ Nontransgenic or injection AD models are often used to study SAD using an intracerebroventricular infusion of streptozotocin (ICV‐STZ). Treatment with this chemical does not contribute to Aβ or P‐tau production but causes significant cognitive impairment likely due to oxidative stress.^49^ Unfortunately, the trend remains the same, and none of the models fully recapitulate the pathology of AD as in humans. There are additional disadvantages to using mouse models, one being that their brains do not function identically to human brains. Mice and humans share about 85% of their genomes, leaving a 15% translational gap that may include substantial differences. Also, human brains consist of roughly an equal proportion of neurons (~1.38 billion neurons)^50^ and glial cells, while mouse brains are roughly 70% neurons (~70 million neurons) and 30% glia.^51^ These differences will impact how AD progresses within mice and human brains.

In addition to mice, nonhuman primates including rhesus monkeys (*Macaca mulattas*), stump‐tailed macaques (*M*. *arctoides*), mouse lemurs (*M*. *murinus*), common marmosets (*C*. *jacchus*), and cynomolgus monkeys (*M*. *fascicularis*) have been used to study SAD. These models can be advantageous for studying naturally aged primates as they more closely resemble the aged, human brain. For example, nonhuman primate models have shown tau protein accumulation as well as Aβ deposits.^52^, ^53^, ^54^, ^55^, ^56^ Cognitive deficits linked to AD symptoms are also observed in nonhuman primate models. While this is extremely relevant to studying AD pathogenesis, there are still key differences in AD pathology between nonhuman primates and humans. In humans, the hippocampus is the main region where Aβ plaques are deposited. However, in rhesus macaques and common marmosets, plaques tend to accumulate in the limbic cortex and temporal cortex, respectively.^53^, ^55^ P‐tau also tends to accumulate in the hippocampus in humans while P‐tau accrues in the cerebral cortex in mouse lemurs.^52^ Additionally, humans can display AD pathology without cognitive impairment, but both aspects are typically seen together in stump‐tailed macaques.^56^ In sum, nonhuman primate models can prove to be advantageous due to natural and similar AD progression to humans, but the pathological differences, the lack of samples due to longer lifespans, and the high costs associated with them cannot be ignored. And, as with all animal models, there are ethical concerns with testing and even more so when disease factors must be introduced.

### 3.2 2D culture models

Two‐dimensional (2D) in vitro cell cultures have been used in research for over a century.^57^ They consist of cells grown in cell culture wells or trans‐wells (Figure 3b) usually coated with compounds such as fibronectin, laminin, and collagen which allow for better cell adhesion and support differentiation.^58^ The key advantages of 2D models include simplicity, low cost, and high‐throughput screening. Unfortunately, 2D models are not suitable for modeling the complexities of AD pathology as they lack the structure and function of the native brain. Despite those shortcomings, typical AD pathologies such as the formation of Aβ plaques and NFTs have been replicated in 2D models.^6^, ^7^, ^8^ Yagi et al. created an induced pluripotent stem cell (iPSC)‐derived neuronal cell culture with PSEN1 and PSEN2 mutations to model FAD and observed increased production of the Aβ42 isomer.^6^ Treating the cells with γ‐secretase also demonstrated potential for drug delivery experiments. Sproul et al. developed an iPSC neuron model from fibroblasts of healthy and FAD patients and observed higher ratios of Aβ42 to Aβ40 in the FAD model as compared to the control.^7^ These models do not fully recapitulate AD due to a lack of NFT pathology. Hossini et al. created an iPSC model using a sample from a SAD patient that contained P‐tau but was missing plaque formation.^8^ 2D cultures serve as simplified systems to characterize AD, but their accuracy in modeling AD is surpassed by 3D cultures and cerebral organoids.

### 3.3 3D culture models

The formation of 3D cultures using hydrogels as extracellular support introduced advancements with in vitro modeling, including the formation of neurospheroids which are a cluster of brain cells that provide some brain‐related function. Unlike cerebral organoids, neurospheroids cannot self‐assemble, lack cellular diversity, and do not contain tissue architecture that resembles in vivo brain formation, but they still serve as useful systems for modeling pathologies.^59^ For example, Choi et al. produced one of the earliest FAD‐based, neurospheroid models using neuronal cell clusters housed in Matrigel. When grown as 3D clusters rather than 2D cultures, results displayed greater aggregation of Aβ and P‐tau due to the limited diffusion of these proteins to surrounding media.^9^, ^60^ The presence of the Matrigel was also noted to slow outward protein diffusion, allowing more time for intracellular accumulation of Aβ and P‐tau. Lee et al. investigated the relationship between BACE1 and γ‐secretase inhibitors and Aβ buildup using neurospheroids cultured from AD patient‐derived iPSCs.^10^ Using APP and PSEN1 mutated human neural progenitor (hNPCs) spheroids, Kwak et al. displayed increased Aβ 42/40 ratio, increased P‐tau, and NFT formation.^11^

The integration of microfluidic‐based platforms for culture systems provides certain advantages in 3D culturing such as modeling blood flow, investigating interactions between spheroids, and understanding the effects of multiple organ systems (Figure 3c). Park et al. introduced a 3D microfluidic device incorporating Matrigel‐filled chambers for housing APP and PSEN1‐mutated neuron/astrocyte cultures with microglia.^12^ In contrast to their 2D microfluidic system, the 3D system contained increased Aβ42 aggregation and P‐tau formation. However, additional experiments also showed migration of microglia to induce neuron/astrocyte loss via IFN‐γ and TLR4 mechanisms. Microglia were also found to substantially increase the production of TNF‐α and nitric oxide (NO) inflammatory markers that contribute to these mechanisms.^12^ Jorfi et al. produced neural spheroids in microwell arrays, and similarly, clusters demonstrated overexpression of APP and PSEN1 after 9 weeks. Interestingly, the spheroids contained dendrites extending outward rather than inward, suggesting this model could be used to study signaling networks between adjacent spheroids.^13^ Tunesi and colleagues took the first steps toward designing a multi‐organ‐on‐a‐chip device to recapitulate the microbiota–gut–brain axis (MGBA) and evaluate its influence on AD pathology. Their platform was demonstrated to be suitable for hydrogel‐based cultures and allowed perfusion of media that mimicked mechanical cues necessary for stimulating certain cell behaviors. Additionally, their device allowed for interconnected units to model the dependency among multiple organ systems. Their findings revealed increased Aβ42 production in APP‐mutated H4 neuroglioma cells using their microfluidic platform.^61^

In addition to modeling AD, cerebral organoids and chip‐based models have been used for drug delivery, efficacy testing, and toxicity assessments for several cognitive diseases.^62^, ^63^, ^64^, ^65^ For example, Groveman et al. created an iPSC‐derived cerebral organoid to model Creutzfeldt–Jakob disease (CJD) as a drug testing platform. In their study, the authors validate the use of an organoid model for CJD by treating organoids with pentosan polysulfate (PPS), an established anti‐prion compound. While the infection was observed to rebound after removal of the PPS treatment, the treated organoids, overall, displayed 10‐fold less prion seeding activity, indicating treatment effectiveness.^62^ Using iPSCs, Park et al. created 1300 cerebral organoids from PiB‐positive and PiB‐negative SAD patients and CRISPR‐Cas9‐edited APOE4 isogenic lines derived from an APOE3 parent line. Activity levels of Aβ plaque formation, P‐tau formation, and synapse loss as a function of oxidative stress were analyzed. Mathematical models to represent the progression rates of these pathologies were generated to identify FDA‐approved drugs that may be repurposed for addressing AD‐related symptoms.^63^ In another example, Renner et al. aimed to improve scalability and automation of cerebral organoid development and high‐throughput drug screening using 96‐well plates. The produced midbrain organoids displayed homogeneous morphology, size, cell composition, and global gene expression, therefore, demonstrating a higher level of standardization and introducing less bias for drug screening.^64^

Cerebral organoid models offer advantages over animal models and 2D cultures in modeling AD (Figure 3d). As research to unravel AD mechanisms continues to surge, cerebral organoids have been gaining popularity as a valuable platform to model this disease. Derived from embryonic stem cells (ESCs) or iPSCs, cerebral organoids are miniature, 3D structures of brain tissue modeling region‐specific anatomy, gene expression, and function.^66^ They are highly sought after in modern research and are likely to mimic human brain pathology and disease pathways more accurately and ethically. Cerebral organoids have also been shown to exhibit significant disease pathology along with a time scale of several months. While animal models can be used to study behavioral changes with disease progression, and 3D neurospheroids offer glimpses into mirroring AD characteristics, they lack the structural and functional accuracy of AD seen in current organoid models.

## 4 TISSUE‐ENGINEERED BRAIN ORGANOIDS FOR MODELING AD

### 4.1 Fabrication of organoids

Assessing brain pathologies using 2D cultures or animal models has unavoidable complications. Cerebral organoid cultures hold promise to fill the gap between 2D models and animal models as the next generation of disease modeling. Earlier stem cell strategies implemented the formation of neural rosettes and precursor neuroepithelial (NE) structures for spatial differentiation into brain domains^67^ by interfering with pathways known to influence neural induction such as SMAD, sonic hedgehog (SHH), and notch signaling.^68^, ^69^

**FIGURE 4:**
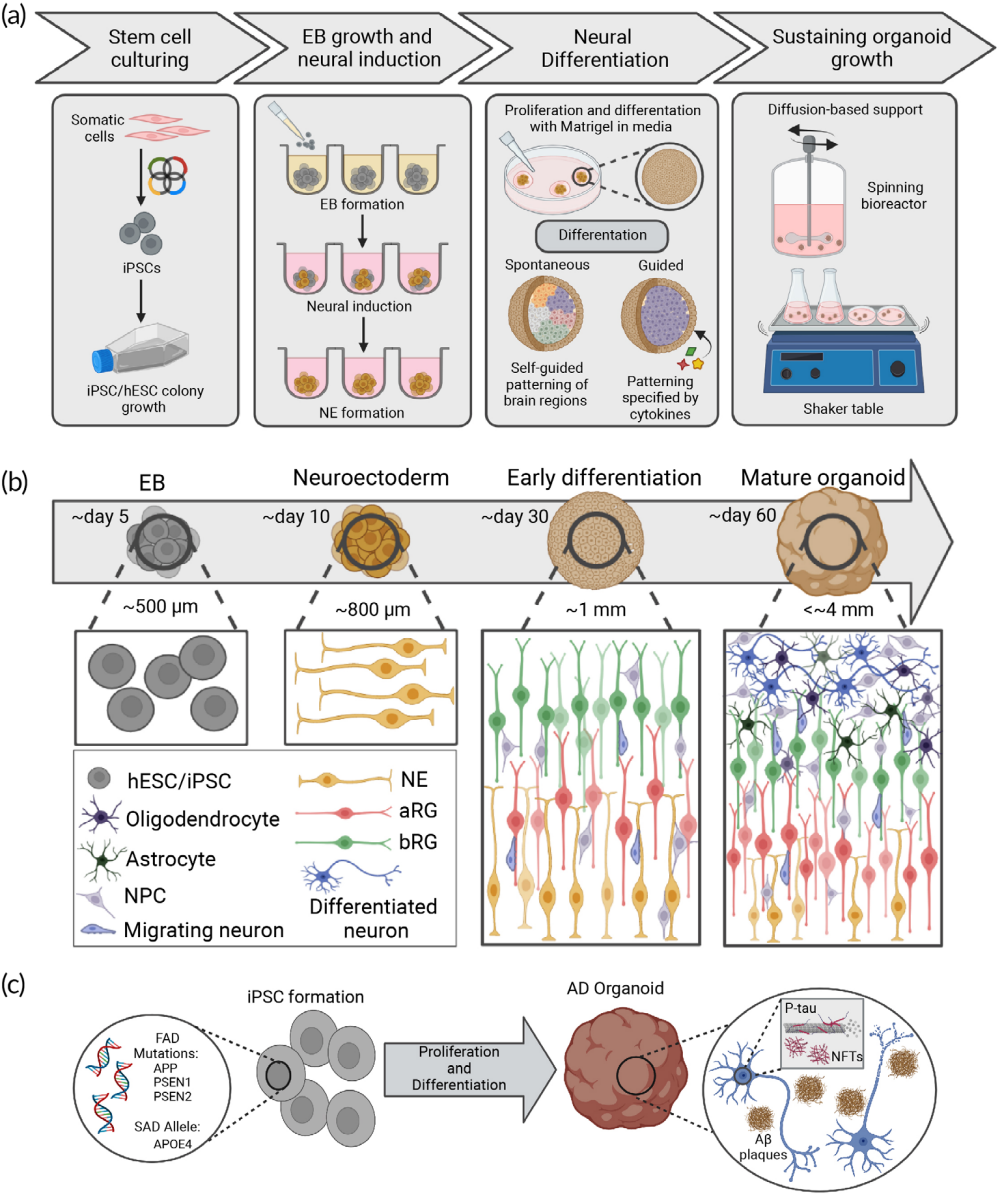
Engineering brain organoids for modeling AD. (a) Schematic illustration of general processes for engineering cerebral organoids from stem cell culturing, EB and NE formation, NE expansion and differentiation, and maintaining organoid viability. The third panel notes varying differentiation tactics to produce either spontaneously‐formed or guided organoids. (b) Development of brain organoids at different stages with approximate sizes and time for formation. Stages are accompanied by diagrams illustrating cell types found alongside their distribution found within the developing organoid c. Engineering AD cerebral organoids from iPSCs with AD mutations. iPSCs carrying FAD mutations or the SAD genetic risk factor, APOE4 allele, are used to generate AD organoids that mimic mechanisms of Aβ plaques and NFT formation found in AD patients. Created with BioRender.com

Figure 4a shows the key processes for developing cerebral organoids, including stem cell culturing, embryoid body (EB) growth, neural induction, neural differentiation, and sustaining organoid growth. Lancaster et al. are credited for the development of self‐organized cerebral organoids containing distinguishable, compartmentalized regions such as the neocortex, hippocampus, and amygdala without the incorporation of exogenic cues.^70^ They list detailed protocols now widely used by groups aiming to produce cerebral organoids. In brief, 5000 iPSCs are seeded into the round bottom, ultra‐low attachment 96‐well plates to form EBs supported with an appropriate medium for growth. After 6 days, EBs are transferred to low‐attachment 24‐well plates containing neural induction medium to form NE tissue over the next 5 days. Tissues are then individually transferred to separate Matrigel droplets. After Matrigel polymerization, the droplets are supplemented with a differentiation medium in stationary culture conditions for 4 days. Afterward, Matrigel droplets containing tissues are placed in a spinning bioreactor with a differentiation medium to promote long‐term organoid viability by encouraging the diffusion of oxygen, nutrients, and wastes.

Spontaneous cerebral organoid maturation resembles endogenous brain development during embryogenesis.^71^ The representative cellular structures of cerebral organoids through their development are shown in Figure 4b. EBs initially created by iPSC seeding form neuroectoderm structures consisting of NE when under the influence of neural induction medium. This forms the basis of the ventricular zone (VZ) along the apical side of the tissue and mimics the foundation seen along the apical surface in the neural tube in vivo.^72^ When introduced to extracellular matrix (ECM) substrates, such as Matrigel, and differentiation medium, NE further proliferates and differentiate into preliminary neural identities such as apical and basal radial glia (aRG, bRG), NPCs, and neurons.^73^ Culturing organoids in spinning bioreactors have shown to promote nutrient and oxygen diffusion in organoids, fostering radial NE growth to produce larger tissue structures.^70^, ^74^ Interkinetic nuclear (IKN) migration of glia and neurons toward the basal side of the neuroectoderm allows for further proliferation, radial expansion, and stratification of cell populations into the VZ, inner and outer subventricular zone (ISVZ and OSVZ), and the cortical plate.^72^ Further differentiation of glial cells yields other specialized neural populations such as oligodendrocytes and astrocytes which also travel towards the basal side via IKN movement.^71^ To produce AD‐associated models, iPSCs containing FAD‐based genetic mutations or the SAD onset risk factor, the APOE4 allele, can be generated from AD patient cells or introduced using gene‐editing techniques such as CRISPR/Cas9.^75^ The resulting organoids can mimic hallmark AD characteristics such as Aβ plaques and NFTs (Figure 4c) and can be used to reveal unknown cellular mechanisms of AD progression or serve as platforms for drug development.

**FIGURE 5:**
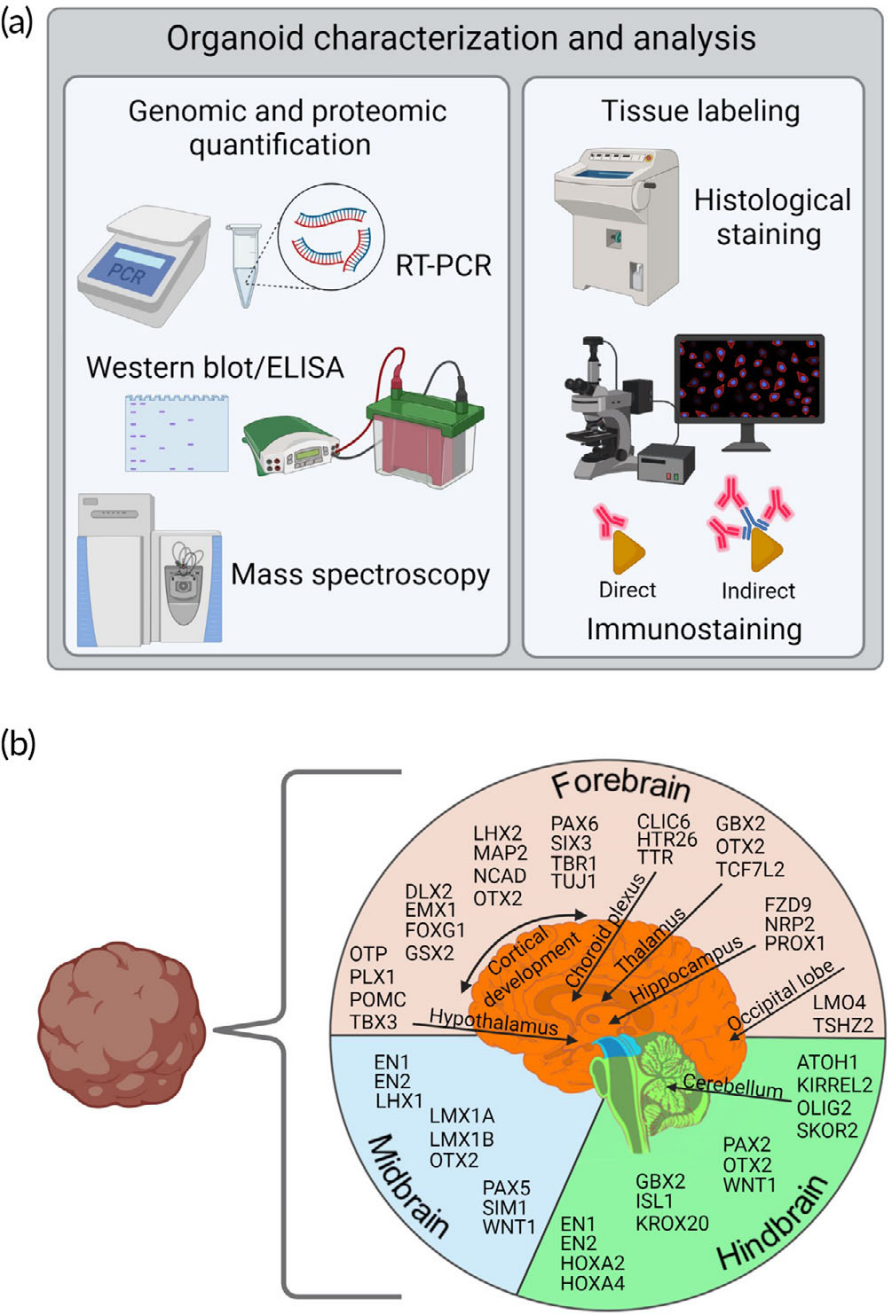
Characterization of brain organoids. (a) Different biotechniques used to characterize the differentiation, compartmentalization, and maturation of cerebral organoids. Panel on the left lists protein detection techniques such as RT‐PCR and western blot/ELISA used to quantify the expression of target proteins, and mass spectroscopy to sample the organoid proteome. Panel on the right lists methods such as histological stains or antibody‐based labeling used to visually classify tissue for the presence, distribution, and quantity of specific proteins. (b) Outline of common proteins previously quantified to distinguish the presence of certain brain regions within cerebral organoids. Proteins are listed based on regions they are associated with and their approximate location within the forebrain, midbrain, or hindbrain. Created with BioRender.com

Based on positioning along the dorsal–ventral and anterior–posterior axes, cells are exposed to varying gradients of signaling factors that dictate cell fate in a concentration‐dependent manner to eventually develop differentiated brain regions. For example, the SHH gradient is a significant guide to inducing ventral‐positioned progenitors to motor neurons,^75^, ^76^ and bone morphogenic proteins (BMPs) instruct dorsal populations along the neural crest for forebrain development.^77^, ^78^ Throughout organoid formation, gene expression and protein quantification/visualization techniques such as immunofluorescence, RT‐PCR, and western blotting techniques are used to confirm maturation, compartmentalization, and the presence of specialized cell populations (Figure 5a). The development of identifiable brain regions is demonstrated by gene expressions comparable to those observed in vivo. For example, transcription factors PAX6 and FOXG1 are commonly tested as forebrain markers while KROX20 and PAX2 detection is utilized to corroborate hindbrain identity.^70^, ^79^, ^80^ Figure 5b maps additional markers used to identify specific regions in the forebrain, midbrain, and hindbrain to corroborate their development and presence in spontaneously developed cerebral organoids.^70^, ^81^, ^82^, ^83^, ^84^, ^85^

A common point of debate involves the use of exogenous signaling cues to optimize cerebral organoid growth. While self‐assembling organoids show discrete brain regionalization, there are inconsistencies reported. Lancaster et al. describe “batch‐to‐batch” variability in EBs depending on the age and morphology of the pluripotent cells used and varying sizes of NE tissue when cultured in Matrigel droplets.^86^ Other researchers such as Quadrato et al. observed sparse populations in cerebral organoid tissue to exhibit non‐neuronal, more mesoderm‐based characteristics through single‐cell RT‐qPCR mapping^87^, ^88^ or contain underdeveloped regions lacking structural maturity or cell diversity. Therefore, strategies that incorporate more guided differentiation methods were developed to address these issues. For example, to encourage higher mesoderm‐derived microglial populations which play crucial roles in AD and autism, methods involving adjusted heparin concentrations and delayed Matrigel embedding during organoid development have been implemented and influenced future studies for microglia promotion.^89^, ^90^, ^91^ Through guided mediation, other studies focused on optimizing discrete regions such as the neocortex, hippocampus, hypothalamus, cerebellum, and choroid plexus to study and manipulate their development and associated transcription profiles (as reviewed in References 92, 93, 94).

Overall, cerebral organoids demonstrate themselves as more sophisticated models in comparison to the 2D culture or animal models as previously described and provide higher accuracy for modeling neurodegenerative diseases. Alongside AD, organoids have been used to model Parkinson's,^95^, ^96^ Huntington's,^97^, ^98^ and CJD.^99^

### 4.2 Genetically induced FAD models

**FIGURE 6:**
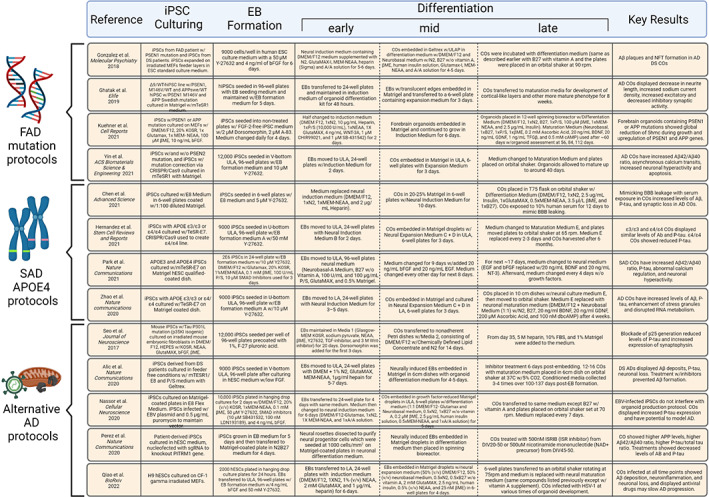
Summary of key protocols for engineering AD brain organoids. Previously used methods and compounds are listed based on FAD mutations, SAD APOE4‐related, and alternative mechanisms related to AD. Steps are separated based on common organoid formation stages such as EB formation and differentiation. Created with BioRender.com

As mentioned previously, FAD is a hereditary form of AD with mutations of APP, PSEN1, and PSEN2, leading to Aβ buildup (specifically a higher ratio of Aβ42 to Aβ40), greater P‐tau and NFT deposition, and ROS release (Figure 2).^100^ After the introduction of cerebral organoids from stem cells containing one or more of these inherited mutations, FAD models have been extensively used to examine pathologies.^9^, ^11^, ^12^, ^13^, ^61^, ^101^, ^102^, ^103^, ^104^, ^105^, ^106^, ^107^ Figure 6 summarizes the protocols for engineering different AD organoids and their key results. Choi and colleagues are credited with developing one of the earliest FAD 3D culture systems by overexpression of APP and PSEN1 that led to the greater aggregation of Aβ and P‐tau.^9^ These results, alongside Lancaster et al.'s development of spontaneous cerebral organoids, paved the road for modeling AD pathology using cerebral organoids.

AD organoids developed from patient‐derived iPSCs provided advances in further recapitulating AD pathology in vitro. Raja et al. introduced a spontaneous cerebral organoid model using patient‐derived iPSCs containing APP or PSEN1 mutations. Alongside abnormal endosome sizes, Aβ aggregation in FAD groups was noticed to be greater on day 60 and day 90 of organoid maturation in comparison to the control, while the heightened presence of P‐tau was seen only on day 90. The results suggest that this model demonstrates an age‐dependency with initial Aβ deposition to eventually produce P‐tau, a mechanism noted with in vivo AD.^101^, ^108^ Gonzalez et al. also utilized patient‐derived iPSCs to produce FAD cerebral organoids containing a PSEN1 mutation and other neurodegenerative models. Similar observations with Aβ and P‐tau deposits were noted with FAD and down syndrome (DS) structures, also displaying a timeline‐like dependency with the formation of P‐tau and the proportionate amount of NFTs.^102^

Aside from emulating the hallmark Aβ and P‐tau pathologies, progress has been made to inspect additional complications found with in vivo FAD. This includes hyperexcitability of neurons, disruptions in calcium (Ca^2+^) signaling dynamics, and alterations in DNA base structures. Ghatak et al. explored the role of hyperexcitability which often leads to synapse loss in AD patients. Cerebral organoids consisting of iPSC‐derived neurons with PSEN1/APP mutations displayed hyperactive synapse firing.^104^ This was shown with increased expression of VGLUT1, a crucial protein that encases glutamates in synaptic vessels for neurotransmission.^109^ This was linked to the shortened dendrites observed in the neural network, and results were found to be consistent with previous hyperexcitability results seen in previous AD animal models.^110^, ^111^ Recently, Yin et al. showed inconsistent Ca^2+^ spikes in organoids derived from iPSCs carrying PSEN2 mutations.^107^ Alongside increased Aβ42, this model displayed other pathological predecessors of AD development such as increased levels and asynchronous exchanges of Ca^2+^.^112^ AD organoids also showed greater hyperactivity with Ca^2+^ firing and were consistent with results reported by Ghatak et al.^104^ Separately, Kuehner et al. showed the influence of DNA 5‐hydroxymethylcytosine (5hmC) levels in normal versus FAD forebrain organoids containing PSEN1 or APP mutations over 112 days.^106^ The presence of 5hmC during embryonic brain development correlates to maturation through neural differentiation, cell functions, and structural development. AD organoids showed an overall reduction of 5hmCs through the 112‐day period and upregulated genes associated with AD, therefore demonstrating the potential onset of FAD due to a lack of 5hmC during fetal brain development.^106^ The timeline‐like dependency seen in other studies was also modeled here to mark the progression of embryo development in terms of DNA structural changes with 5hmC levels.

### 4.3 Genetically induced SAD models

SAD has been considered more difficult to model due to the lack of associated mutations and the influence of external stimuli,^113^ yet, it consists of similar pathologies associated with FAD such as Aβ accumulation, P‐tau, NFT formation, and neuronal loss (Figure 2). Symptoms in SAD patients are typically seen later than in patients diagnosed with FAD and its onset is strongly linked to APOE4.^18^ Therefore, many established models of SAD organoids utilize iPSCs derived from AD APOE4 carriers or use CRISPR/Cas9 to edit genomes to contain the ε4 isoform.^114^, ^115^, ^116^, ^117^, ^118^

Lin et al. used CRISPR/Cas9 to edit the APOE3 in iPSCs to the APOE4 allele and generate independent cultures of neurons, microglia, and astrocytes.^114^ In comparison to the control, neurons expressed increased synapse activity and 20% higher Aβ42 levels. Also, microglia had reduced Aβ42 phagocytic activity and increased inflammatory expression, and astrocytes also displayed reduced efficiency in clearing Aβ42. Cerebral organoids containing the APP mutation and Aβ42 aggregation were co‐cultured with APOE4 microglia and results demonstrated increased Aβ42 formation and P‐tau. Additionally, 6‐month SAD patient‐derived organoids with APOE4 to APOE3 converted genomes expressed reduced Aβ42, highlighting the influence of APOE4 in further promoting AD.^114^ Zhao and colleagues investigated multiple AD progression pathways using healthy and AD patient‐derived iPSCs containing homozygous APOE3 or APOE4 allele types. Their results confirmed the synergistic repercussions of APOE4 presence with increased neuronal apoptosis, synapse loss, and P‐tau mainly in AD organoids after 12 weeks. Interestingly, Aβ aggregation was not significant in AD organoids. Some pathology characteristics in SAD tend to appear later than FAD^101^ which may explain the aggregation seen with Lin et al.'s 6‐month organoids versus its absence in Zhao et al.'s 12‐week organoids. However, in alignment with Lin et al., SAD patient‐derived organoids with APOE4 to APOE3 editing showed lower Aβ formation.^116^ Recently, Hernandez and colleagues showed almost no influence by APOE status with insignificant Aβ production in healthy organoids containing homozygous APOE3 or APOE4.^115^ P‐tau levels were unchanged except at the S199 phosphorylation site. While these results do not confirm pathological initiation by APOE4, it emphasizes APOE4's additive influence on pathology traits with AD‐defined cultures. The authors also state the contrasting results to Lin et al. could be due to varying cell cultures used.

Several SAD models also examined the hallmark characteristics of the BBB. For example, Blanchard et al. studied the contribution of APOE4 to cerebral amyloid angiopathy, the deposition of Aβ in cerebral blood vessels leading to hemorrhage, and neurovascular disorders such as AD.^119^ The authors mimic BBB structure and behavior using a self‐assembled tri‐culture of brain endothelial cells, mural cells, and astrocytes in Matrigel. All cell lineages were derived from iPSCs with homozygous APOE3, APOE4, or a heterozygous combination. The highest levels of Aβ were produced by BBBs developed from homozygous APOE4 followed by the heterozygous combination.^118^ Similarly, Chen et al. produced a cortical organoid model to observe BBB leakage typically observed in SAD. As cerebral organoids currently lack vasculature and blood flow, BBB leakage was emulated by exposing the organoid to human serum for 12 days. As hypothesized, Aβ accumulation increased in serum‐treated organoids through increased expression of beta‐secretase 1 (BACE), one of the proteins responsible for cleaving APP to form Aβ aggregates. Additionally, higher P‐tau was also observed via the GSK3α/β tau protein‐kinase mediated pathway.^120^

### 4.4 Alternative mechanisms used for AD models

Additional studies invoke AD pathology by exploring alternative signaling pathways instead of manipulating genes directly associated with FAD or SAD.^121^, ^122^, ^123^, ^124^ For example, a carrier of homozygous APOE3 may still develop SAD due to influences of environmental stimuli. To model this circumstance, Pavoni et al. introduced a chemically‐induced AD model by using the compound Aftin‐5 to upregulate Aβ42 production in healthy patient‐derived organoids.^121^ This inducer works to limit β‐secretase or γ‐secretase inhibitors.^125^ Cairns et al. explored the involvement of herpes simplex virus type 1 (HSV‐1) in promoting hallmark AD traits in neural spheroids grown in porous silk scaffolds. Past studies indicated the involvement of pathogens like HSV‐1 in further developing SAD^126^; while HSV‐1 is typically latent, its activated form can infiltrate the BBB. After 3 days of infection, increased Aβ plaques were seen alongside P‐tau and NFT formation, reactive gliosis, expression of PSEN1 and PSEN2, and neuroinflammation. This further underlines the link between HSV‐1 infection and AD behaviors.^123^ A recent study conducted by Qiao et al. using HSV‐1 infected organoids demonstrated similar results with Aβ deposition, neuroinflammation, and neuronal loss with reversed effects after exposure to antiviral drugs Ribavirin and Valacyclovir.^127^ Separately, Perez et al. found the inhibition of pitrilysin metallopeptidase 1 (PITRM1), a mitochondrial protease previously found to degrade Aβ peptides, increased Aβ aggregation, P‐tau, and neuronal apoptosis of iPSC‐derived cortical and cerebral organoids. Additionally, its upregulation of mitochondrial unfolded protein response (UPR^mt^) may lead to downstream APP and Aβ42 accumulation,^124^ therefore, indicating mitochondrial factors might also play a hand in AD characteristics.

Other researchers introduced novel AD platforms or methods to broaden disease analysis,^128^, ^129^, ^130^ Chen et al. provided the first proteomic filing for neurospheroids using mass spectroscopy. Protein expression changes linked to axon development, gliogenesis, and immune response were found to mimic those found in post‐mortem AD tissue. While the iPSC‐derived neurospheroids contained identical genomes to those found in post‐mortem tissues, the neurospheroids were not associated with any particular brain region while the post‐mortem tissue was specifically removed from the frontal cortex, interior cortex, and cerebellum.^128^ Therefore, more proteomic filing studies would shed light on identifying varying expression profiles for AD among brain regions. In another example, Cai et al. used acoustofluidics technology to assemble neurospheroids containing neurons, Aβ clusters, and microglia to model AD. Similar to in vivo conditions, cultures displayed increased toxicity, inflammation, and microglia migration toward the Aβ aggregates.^130^ Overall, these alternative models provide their unique advantages and mechanisms to similarly characterize AD without the genetic modifications associated with FAD or SAD.

## 5 PERSPECTIVE

### 5.1 Engineering brain organoids with vasculature

Although cerebral organoids have been proposed as a superior alternative to animal models, they come with their own set of limitations. One issue that greatly hinders the growth potential of organoids is the lack of overlying meninges and vasculature. These structures provide support for brain development and their absence creates batch‐to‐batch variation.^86^ The lack of vasculature in cerebral organoids is concerning for several reasons. Without a significant vascular system, the organoid is entirely dependent on diffusion from its extracellular matrix for oxygen and nutrients. As the organoid grows, diffusion is no longer sufficient for transporting enough oxygen and nutrients to the center of the organoid, leading to necrosis and eventually rendering the organoid unsuitable for experimentation.^131^ This poses a challenge for modeling AD in organoids due to a lack of aging in comparison to in vivo AD brains. Instead, the cerebral organoids more closely resemble a fetal brain. Because this issue has not been fully resolved, it is difficult to estimate the time duration necessary to better recapitulate AD pathology as seen in the adult human brain. Spinning bioreactors are one way of increasing homogeneity across organoids via enhanced diffusion of oxygen and nutrients without adding vasculature. This method reduces necrosis and promotes larger cortical structures.^74^, ^86^ Mansour et al. acknowledged the issue of vascularization by grafting a brain organoid onto mice brains to promote blood vessel formation in vivo. Although immunofluorescence staining and confocal microscopy confirmed that the graft was significantly vascularized, there was no data provided on the graft once it is removed, that is, if they continue to survive in vitro.^131^ This method works to vascularize organoids, yet it may be considered counterintuitive since animals are still used to create in vitro models meant to replace the need for in vivo testing.

Coupling angiogenesis with organoid formation, precasting vasculature structure in organoid models, and 3D printing vasculature all have the potential for engineering brain organoids with vasculature. Angiogenesis is the key step for the vascularization of the brain during embryogenesis. By coupling angiogenesis with neurogenesis during organoid formation, we can theoretically generate perfusable cerebral organoids that can grow beyond the diffusion limits. However, optimized specifications, such as growth factors, growth factor concentrations, ECM, flow properties, and timings for cell differentiation, are needed. An example of this can be seen in a study by Shin et al. with vascularizing neurospheroids within a microfluidic chip. However, the authors noted the chip cannot be used for vascularizing organoids.^132^ Another potential solution is to precast empty channels in the brain organoids at the early stage and seed those channels with endothelial cells as brain blood vessels. Finally, the rapid development of 3D printing systems will likely enable the engineering of different vascularized tissue, including cerebral organoids, in the near future.

### 5.2 Introducing cellular diversity

Another limitation of current cerebral organoid models is the lack of cell diversity, especially the lack of microglia. Aβ plaques have been shown to stimulate microglia, causing inflammatory responses that contribute to neuronal loss and damage to synapses (Figure 2d). Microglia also participate in the process of degrading Aβ plaques and thus are essential for modeling AD pathology.^36^, ^37^ Culturing differentiated microglia with a maturing organoid is one approach to address this issue. Another method is to grow primitive macrophage progenitors (PMPs) with NPCs at the beginning stages of organoid growth, as done by Xu et al.^133^ As the organoid matures, the surrounding progenitors will differentiate concomitantly. This strategy also allows for control over the ratio of microglia to neurons for more homogeneous organoids that match the ratio seen in vivo. Song et al. co‐cultured microglia‐like cells with dorsal–ventral organoids, and phase‐contrast images overlapped with fluorescent images showed integration of the microglia into the organoids. They were able to use their model to study the inflammatory responses of the microglia when exposed to Aβ plaques.^134^ Nzou et al. also addressed the problem of cell diversity by co‐culturing endothelial cells, pericytes, astrocytes, oligodendrocyte progenitor cells, microglia, and iPSC‐derived cerebral organoids separately. Once the desired number of non‐neuronal cells was reached, cells were harvested and cultured alongside the organoid. This process created a distinct BBB, and its functionality was tested through treatment with mercury ions. The organoids with the BBB demonstrated low permeability to the mercury ions in comparison to the control group (no BBB, traditionally grown organoids).^135^ Co‐culturing, also seen in other investigations discussed earlier, shows strong promise in creating a more accurate model of the human brain. The method of co‐culturing progenitor/precursor cells better mimics the process of differentiation in vivo, meaning the cellular interactions occurring between the differentiating cells are more relevant and more likely to mirror normal brain development.

Cho et al. cultured cerebral organoids in a decellularized brain ECM taken directly from human patient samples. This method allowed greater cell diversity to develop in the organoid such as the inclusion of a higher microglial population. A more complex structure including several ventricle‐like structures and a thicker cortical layer were also observed.^136^ The more intricate structure suggests using a human brain ECM may provide the support that Lancaster's model was lacking due to missing meninges. This method of growing cerebral organoids seems ideal as they are in a similar environment in which the brain develops in vivo, but even if researchers use human brain samples to replicate this model, they cannot guarantee qualitative consistency across all samples.

### 5.3 Alternatives to Matrigel for generation and maintenance of organoids

Another limitation of organoids is their batch‐to‐batch variability.^137^ The matrix used in culturing organoids is a significant factor contributing to this variability. For example, Matrigel is the most common matrix used for embedding organoids, but Matrigel has a high batch‐to‐batch variability regarding its composition and mechanical properties, and its contents are not well characterized.^138^ Cassel de Camps et al. modified Matrigel with an interpenetrating network (IPN) of alginate to modify the mechanical properties and noted that increasing the stiffness of the matrix led to more mature neuronal phenotypes and affected the size and number of neural rosettes within the organoid.^139^ This exhibits the importance of the embedding matrix and its potential to be used to tune organoid properties. Similarly, Raimondi et al. demonstrated the important role of hydrogels in the growth of different CNS cells by developing collagen‐based semi‐IPN, composed of either hyaluronate or polyethylene glycol (PEG), to grow 3D cultures of neurons, microglia, and astrocytes.^140^ Although they did not use their hydrogels to grow organoids, Raimondi et al. created triple cultures using their synthetic hydrogel to optimize cell ratios.

Using synthetic hydrogels provides more control over the environment in which the cerebral organoids grow and thus allows for less batch‐to‐batch variability. For example, Ranga et al. generated a PEG‐based synthetic hydrogel to grow cerebral organoids.^141^ Compared to cerebral organoids grown in Matrigel, those grown in the synthetic hydrogel were more even, albeit slightly smaller than those grown in Matrigel. Lindborg et al. also generated a synthetic hydrogel composed of sodium hyaluronan and chitosan, termed Cell‐Mate3D.^142^ As the contents of these synthetic hydrogels are defined, they allow for more widespread use of organoids in downstream applications.

Another limitation of using Matrigel is that it does not fully represent the complexity of the CNS environment.^143^ To address this, studies have synthesized hydrogels from decellularized brain ECM. Simsa et al. used a decellularized adult porcine brain ECM (B‐ECM) for growing hESC‐derived brain organoids in comparison with Matrigel.^144^ On day 10 of development, they saw more uniform growth in the organoids grown in B‐ECM hydrogel. However, on day 40, no differences were observed between the two groups. This uniform growth may be beneficial for studying brain development or disease pathogenesis. Cho et al. generated organoids grown in decellularized ECM from human brain tissue, which better mimics the compositions in brain ECM. Organoids were cultured within microfluidic chips to allow for fluid flow to resemble cerebrospinal fluid flow and demonstrate more reproducible results.^136^ Organoids grown in their brain ECMs had enhanced growth, differentiation, cortical layer development, and electrophysiology compared to those grown in Matrigel, but the batch‐to‐batch variability was only improved with the use of their microfluidic chip. Therefore, the development of alternative hydrogels for embedding cerebral organoids is an important area of study.

### 5.4 Multi‐organ models

Although AD pathologies are primarily associated with the central nervous system no tissue or organ in the human body is completely isolated from other organs. Several studies link the gut microbiome, kidneys, and heart to AD pathology, cognitive impairment, and/or brain inflammation.^145^, ^146^, ^147^, ^148^, ^149^, ^150^, ^151^, ^152^, ^153^ A study by Cattaneo et al. recognized that patients suffering from cognitive impairment and brain amyloidosis also had an abundance of the proinflammatory gut microbiome taxon genera *Escherichia* and *Shigella*, and a reduction of anti‐inflammatory taxon *E*. *rectale*. They hypothesized that the composition of the gut microbiome may have some effect on brain inflammation, which would contribute to brain amyloidosis and potentially neurodegeneration.^147^ Zhan et al. studied brain samples from deceased AD patients and found lipopolysaccharides and *E*. *coli* in the brain parenchyma of all samples in higher amounts than in those control samples.^152^ Patients with chronic kidney disease have been shown to have concentrations of urea, nitrogen, and creatinine up to 10 times higher than normal in areas of the brain relating to cognition.^148^ Heart failure or cardiac insufficiency can cause irregular vasoconstriction leading to more narrow microvasculature, effectively reducing the amount of oxygen going to the brain which is well known to cause cognitive impairment.^154^ Although these findings are not directly correlated with AD, they show the need for more complex models involving more than only cerebral organoids to better understand mechanisms of cognitive impairment which is the most abundant symptom of AD. For example, as mentioned previously, Tunesi et al. designed a multi‐organ‐on‐a‐chip platform to model the MGBA for exploring the potential interplay between different organ systems.^61^

## 6 CONCLUSION

Cerebral organoids provide the next‐generation platform for modeling the origins and mechanisms of AD progression and for screening effective treatments. Their ability to capture the development and architecture of human neuronal tissues is unparalleled to previous 2D cultures and animal models, thereby offering a more accurate model system. Drawbacks such as a lack of vasculature, cellular diversity, and a young developmental age will need to be addressed to develop more robust platforms for advanced screening. However, current progress to overcome these limitations provides a bright outlook for producing more conclusive, in‐depth studies for AD and other neurodegenerative diseases.

## AUTHOR CONTRIBUTIONS

**Sai sreenivasamurthy:** Conceptualization (equal); writing – original draft (equal). **Mahek Laul:** Conceptualization (equal); writing – original draft (equal). **Tiffany Kim:** Writing – original draft (supporting); writing – review and editing (supporting). **Nan Zhao:** Conceptualization (equal); writing – original draft (equal); writing – review and editing (equal). **Donghui Zhu:** Conceptualization (equal); project administration (lead); resources (lead); supervision (lead); writing – review and editing (lead).

## CONFLICT OF INTEREST

The authors declared no conflicts of interest for this article.

## Data Availability

Data sharing is not applicable to this article as no new data were created or analyzed in this study.

## References

[1] Matthews KA , Xu W , Gaglioti AH , et al. Racial and ethnic estimates of Alzheimer's disease and related dementias in the United States (2015–2060) in adults aged≥ 65 years. Alzheimer's Dement. 2019;15:17‐24. 30243772 10.1016/j.jalz.2018.06.3063 PMC6333531

[2] Cass SP . Alzheimer's disease and exercise: a literature review. Curr Sports Med Rep. 2017;16:19‐22. 28067736 10.1249/JSR.0000000000000332

[3] Gervès C , Chauvin P , Bellanger MM . Evaluation of full costs of care for patients with Alzheimer's disease in France: the predominant role of informal care. Health Policy. 2014;116:114‐122. 24461717 10.1016/j.healthpol.2014.01.001

[4] Selkoe DJ , Hardy J . The amyloid hypothesis of Alzheimer's disease at 25 years. EMBO Mol Med. 2016;8:595‐608. 27025652 10.15252/emmm.201606210 PMC4888851

[5] Dorszewska J , Prendecki M , Oczkowska A , Dezor M , Kozubski W . Molecular basis of familial and sporadic Alzheimer's disease. Curr Alzheimer Res. 2016;13:952‐963. 26971934 10.2174/1567205013666160314150501

[6] Yagi T , Ito D , Okada Y , et al. Modeling familial Alzheimer's disease with induced pluripotent stem cells. Hum Mol Genet. 2011;20:4530‐4539. 21900357 10.1093/hmg/ddr394

[7] Sproul AA , Jacob S , Pre D , et al. Characterization and molecular profiling of PSEN1 familial Alzheimer's disease iPSC‐derived neural progenitors. PLoS One. 2014;9:e84547. 24416243 10.1371/journal.pone.0084547 PMC3885572

[8] Hossini AM , Megges M , Prigione A , et al. Induced pluripotent stem cell‐derived neuronal cells from a sporadic Alzheimer's disease donor as a model for investigating AD‐associated gene regulatory networks. BMC Genomics. 2015;16:1‐22. 25765079 10.1186/s12864-015-1262-5 PMC4344782

[9] Choi SH , Kim YH , Hebisch M , et al. A three‐dimensional human neural cell culture model of Alzheimer's disease. Nature. 2014;515:274‐278. 25307057 10.1038/nature13800 PMC4366007

[10] Lee H‐K , Velazquez Sanchez C , Chen M , et al. Three dimensional human neuro‐spheroid model of Alzheimer's disease based on differentiated induced pluripotent stem cells. PLoS One. 2016;11:e0163072. 27684569 10.1371/journal.pone.0163072 PMC5042502

[11] Kwak SS , Washicosky KJ , Brand E , et al. Amyloid‐β42/40 ratio drives tau pathology in 3D human neural cell culture models of Alzheimer's disease. Nat Commun. 2020;11:1‐14. 32170138 10.1038/s41467-020-15120-3 PMC7070004

[12] Park J , Wetzel I , Marriott I , et al. A 3D human triculture system modeling neurodegeneration and neuroinflammation in Alzheimer's disease. Nat Neurosci. 2018;21:941‐951. 29950669 10.1038/s41593-018-0175-4 PMC6800152

[13] Jorfi M , D'Avanzo C , Tanzi RE , Kim DY , Irimia D . Human neurospheroid arrays for in vitro studies of Alzheimer's disease. Sci Rep. 2018;8:1‐13. 29402979 10.1038/s41598-018-20436-8 PMC5799361

[14] King A . The search for better animal models of Alzheimer's disease. Nature. 2018;559:S13‐S15. 30046083 10.1038/d41586-018-05722-9

[15] Bubnys A , Tsai L‐H . Harnessing cerebral organoids for Alzheimer's disease research. Curr Opin Neurobiol. 2022;72:120‐130. 34818608 10.1016/j.conb.2021.10.003

[16] Kang YJ , Cho H . Human brain organoids in Alzheimer's disease. Organoid. 2021;1:e5.

[17] Penney J , Ralvenius WT , Tsai L‐H . Modeling Alzheimer's disease with iPSC‐derived brain cells. Mol Psychiatry. 2020;25:148‐167. 31391546 10.1038/s41380-019-0468-3 PMC6906186

[18] Mamun AA , Uddin MS , Bin Bashar MF , et al. Molecular insight into the therapeutic promise of targeting APOE4 for Alzheimer's disease. Oxid Med Cell Longev. 2020;2020:1‐16. 10.1155/2020/5086250 PMC7245681 32509144

[19] Roberts R , Knopman DS . Classification and epidemiology of MCI. Clin Geriatr Med. 2013;29:753‐772. 24094295 10.1016/j.cger.2013.07.003 PMC3821397

[20] McKhann GM , Knopman DS , Chertkow H , et al. The diagnosis of dementia due to Alzheimer's disease: recommendations from the National Institute on Aging‐Alzheimer's Association workgroups on diagnostic guidelines for Alzheimer's disease. Alzheimers Dement. 2011;7:263‐269. 21514250 10.1016/j.jalz.2011.03.005 PMC3312024

[21] Erickson MA , Banks WA . Blood‐brain barrier dysfunction as a cause and consequence of Alzheimer's disease. J Cereb Blood Flow Metab. 2013;33:1500‐1513. doi:10.1038/jcbfm.2013.135 23921899 PMC3790938

[22] Blair LJ , Frauen HD , Zhang B , et al. Tau depletion prevents progressive blood‐brain barrier damage in a mouse model of tauopathy. Acta Neuropathol Commun. 2015;3:8. doi:10.1186/s40478-015-0186-2 25775028 PMC4353464

[23] Zlokovic BV . Neurovascular pathways to neurodegeneration in Alzheimer's disease and other disorders. Nat Rev Neurosci. 2011;12:723‐738. doi:10.1038/nrn3114 22048062 PMC4036520

[24] Du X , Wang X , Geng M . Alzheimer's disease hypothesis and related therapies. Transl Neurodegener. 2018;7:2. doi:10.1186/s40035-018-0107-y 29423193 PMC5789526

[25] Stelzer G , Rosen R , Plaschkes I , et al. The GeneCards suite: from gene data mining to disease genome sequence analyses. Curr Protoc Bioinformatics. 2016;54:1.30.1‐1.30.33. 10.1002/cpbi.5 27322403

[26] Kojro E , Fahrenholz F . The non‐amyloidogenic pathway: structure and function of α‐secretases. Alzheimer's Dis. 2005;38:105‐127. 10.1007/0-387-23226-5_5 15709475

[27] Zhao J , Liu X , Xia W , Zhang Y , Wang C . Targeting amyloidogenic processing of APP in Alzheimer's disease. Front Mol Neurosci. 2020;13:137. 32848600 10.3389/fnmol.2020.00137 PMC7418514

[28] Vassar R . Bace 1. J Mol Neurosci. 2004;23:105‐113. 15126696 10.1385/JMN:23:1-2:105

[29] Burdick D , Soreghan B , Kwon M , et al. Assembly and aggregation properties of synthetic Alzheimer's A4/beta amyloid peptide analogs. J Biol Chem. 1992;267:546‐554. 1730616

[30] Julia T , Goate AM . Genetics of β‐amyloid precursor protein in Alzheimer's disease. Cold Spring Harb Perspect Med. 2017;7:a024539. 28003277 10.1101/cshperspect.a024539 PMC5453386

[31] Vassar R , Bennett BD , Babu‐Khan S , et al. β‐Secretase cleavage of Alzheimer's amyloid precursor protein by the transmembrane aspartic protease BACE. Science. 1999;286:735‐741. doi:10.1126/science.286.5440.735 10531052

[32] Sengupta A , Kabat J , Novak M , Wu Q , Grundke‐Iqbal I , Iqbal K . Phosphorylation of tau at both Thr 231 and Ser 262 is required for maximal inhibition of its binding to microtubules. Arch Biochem Biophys. 1998;357:299‐309. 9735171 10.1006/abbi.1998.0813

[33] Wang JZ , Grundke‐Iqbal I , Iqbal K . Kinases and phosphatases and tau sites involved in Alzheimer neurofibrillary degeneration. Eur J Neurosci. 2007;25:59‐68. 17241267 10.1111/j.1460-9568.2006.05226.x PMC3191918

[34] Metcalfe MJ , Figueiredo‐Pereira ME . Relationship between tau pathology and neuroinflammation in Alzheimer's disease. Mount Sinai J Med. 2010;77:50‐58. 10.1002/msj.20163 PMC2904237 20101714

[35] Rissman RA , Poon WW , Blurton‐Jones M , et al. Caspase‐cleavage of tau is an early event in Alzheimer disease tangle pathology. J Clin Invest. 2004;114:121‐130. 15232619 10.1172/JCI20640 PMC437967

[36] Fan Z , Brooks DJ , Okello A , Edison P . An early and late peak in microglial activation in Alzheimer's disease trajectory. Brain. 2017;140:792‐803. 28122877 10.1093/brain/aww349 PMC5837520

[37] Zuroff L , Daley D , Black KL , Koronyo‐Hamaoui M . Clearance of cerebral Aβ in Alzheimer's disease: reassessing the role of microglia and monocytes. Cell Mol Life Sci. 2017;74:2167‐2201. 28197669 10.1007/s00018-017-2463-7 PMC5425508

[38] Alexander AG , Marfil V , Li C . Use of *Caenorhabditis elegans* as a model to study Alzheimer's disease and other neurodegenerative diseases. Front Genet. 2014;5:279. doi:10.3389/fgene.2014.00279 25250042 PMC4155875

[39] Iijima‐Ando K , Iijima K . Transgenic drosophila models of Alzheimer's disease and tauopathies. Brain Struct Funct. 2010;214:245‐262. doi:10.1007/s00429-009-0234-4 19967412 PMC2849836

[40] Newman M , Ebrahimie E , Lardelli M . Using the zebrafish model for Alzheimer's disease research. Front Genet. 2014;5:189. doi:10.3389/fgene.2014.00189 25071820 PMC4075077

[41] Carlson GA , Borchelt DR , Dake A , et al. Genetic modification of the phenotypes produced by amyloid precursor protein overexpression in transgenic mice. Hum Mol Genet. 1997;6:1951‐1959. 9302276 10.1093/hmg/6.11.1951

[42] Chen G , Chen KS , Knox J , et al. A learning deficit related to age and β‐amyloid plaques in a mouse model of Alzheimer's disease. Nature. 2000;408:975‐979. 11140684 10.1038/35050103

[43] Games D , Adams D , Alessandrini R , et al. Alzheimer‐type neuropathology in transgenic mice overexpressing V717F β‐amyloid precursor protein. Nature. 1995;373:523‐527. 7845465 10.1038/373523a0

[44] Irizarry MC , McNamara M , Fedorchak K , Hsiao K , Hyman BT . APPSw transgenic mice develop age‐related Aβ deposits and neuropil abnormalities, but no neuronal loss in CA1. J Neuropathol Exp Neurol. 1997;56:965‐973. 9291938 10.1097/00005072-199709000-00002

[45] Irizarry MC , Soriano F , McNamara M , et al. Aβ deposition is associated with neuropil changes, but not with overt neuronal loss in the human amyloid precursor protein V717F (PDAPP) transgenic mouse. J Neurosci. 1997;17:7053‐7059. 9278541 10.1523/JNEUROSCI.17-18-07053.1997 PMC6573263

[46] Morgan D , Diamond DM , Gottschall PE , et al. Aβ peptide vaccination prevents memory loss in an animal model of Alzheimer's disease. Nature. 2000;408:982‐985. 11140686 10.1038/35050116

[47] Schwab C , Hosokawa M , McGeer PL . Transgenic mice overexpressing amyloid beta protein are an incomplete model of Alzheimer disease. Exp Neurol. 2004;188:52‐64. 15191802 10.1016/j.expneurol.2004.03.016

[48] Sturchler‐Pierrat C , Abramowski D , Duke M , et al. Two amyloid precursor protein transgenic mouse models with Alzheimer disease‐like pathology. Proc Natl Acad Sci USA. 1997;94:13287‐13292. 9371838 10.1073/pnas.94.24.13287 PMC24301

[49] Salkovic‐Petrisic M , Knezovic A , Hoyer S , Riederer P . What have we learned from the streptozotocin‐induced animal model of sporadic Alzheimer's disease, about the therapeutic strategies in Alzheimer's research. J Neural Transm. 2013;120:233‐252. 22886150 10.1007/s00702-012-0877-9

[50] Azevedo FA , Carvalho LR , Grinberg LT , et al. Equal numbers of neuronal and nonneuronal cells make the human brain an isometrically scaled‐up primate brain. J Comp Neurol. 2009;513:532‐541. 19226510 10.1002/cne.21974

[51] Erö C , Gewaltig M‐O , Keller D , Markram H . A cell atlas for the mouse brain. Front Neuroinform. 2018;12:84. 30546301 10.3389/fninf.2018.00084 PMC6280067

[52] Bons N , Mestre N , Ritchie K , Petter A , Podlisny M , Selkoe D . Identification of amyloid beta protein in the brain of the small, short‐lived Lemurian primate Microcebus murinus. Neurobiol Aging. 1994;15:215‐220. doi:10.1016/0197-4580(94)90115-5 7838294

[53] Maclean CJ , Baker HF , Ridley RM , Mori H . Naturally occurring and experimentally induced β‐amyloid deposits in the brains of marmosets (Callithrix jacchus). J Neural Transm. 2000;107:799‐814. doi:10.1007/s007020070060 11005545

[54] Nakamura S , Nakayama H , Goto N , Ono F , Sakakibara I , Yoshikawa Y . Histopathological studies of senile plaques and cerebral amyloidosis in cynomolgus monkeys. J Med Primatol. 1998;27:244‐252. doi:10.1111/j.1600-0684.1998.tb00244.x 9926980

[55] Sani S , Traul D , Klink A , et al. Distribution, progression and chemical composition of cortical amyloid‐β deposits in aged rhesus monkeys: similarities to the human. Acta Neuropathol. 2003;105:145‐156. doi:10.1007/s00401-002-0626-5 12536225

[56] Toledano A , Álvarez MI , López‐Rodríguez AB , Toledano‐Díaz A , Fernández‐Verdecia CI . Does Alzheimer disease exist in all primates? Alzheimer pathology in non‐human primates and its pathophysiological implications (II). Neurología (English Ed). 2014;29:42‐55. doi:10.1016/j.nrleng.2011.05.006 21871692

[57] Harrison RG . Observations on the living developing nerve fiber. Proc Soc Exp Biol Med. 1906;4:140‐143.

[58] Hazel T , Müller T . Culture of neuroepithelial stem cells. Curr Protoc Neurosci. 1997;1:3.1.1‐3.1.6. 10.1002/0471142301.ns0301s00 18428459

[59] Zhuang P , Sun AX , An J , Chua CK , Chew SY . 3D neural tissue models: from spheroids to bioprinting. Biomaterials. 2018;154:113‐133. 29120815 10.1016/j.biomaterials.2017.10.002

[60] Li H , Wijekoon A , Leipzig ND . 3D differentiation of neural stem cells in macroporous photopolymerizable hydrogel scaffolds. PLoS One. 2012;7:e48824. 23144988 10.1371/journal.pone.0048824 PMC3492243

[61] Tunesi M , Izzo L , Raimondi I , Albani D , Giordano C . A miniaturized hydrogel‐based in vitro model for dynamic culturing of human cells overexpressing beta‐amyloid precursor protein. J Tissue Eng. 2020;11:2041731420945633. 32922719 10.1177/2041731420945633 PMC7446262

[62] Groveman BR , Ferreira NC , Foliaki ST , et al. Human cerebral organoids as a therapeutic drug screening model for Creutzfeldt‐Jakob disease. Sci Rep. 2021;11:5165. doi:10.1038/s41598-021-84689-6 33727594 PMC7943797

[63] Park JC , Jang SY , Lee D , et al. A logical network‐based drug‐screening platform for Alzheimer's disease representing pathological features of human brain organoids. Nat Commun. 2021;12:280. doi:10.1038/s41467-020-20440-5 33436582 PMC7804132

[64] Renner H , Grabos M , Becker KJ , et al. A fully automated high‐throughput workflow for 3D‐based chemical screening in human midbrain organoids. Elife. 2020;9:e52904. 33138918 10.7554/eLife.52904 PMC7609049

[65] Cvetkovic C , Patel R , Shetty A , et al. Assessing Gq‐GPCR‐induced human astrocyte reactivity using bioengineered neural organoids. J Cell Biol. 2022;221:e202107135. 35139144 10.1083/jcb.202107135 PMC8842185

[66] Lancaster MA , Knoblich JA . Organogenesis in a dish: modeling development and disease using organoid technologies. Science. 2014;345:1247125. 25035496 10.1126/science.1247125

[67] Wilson PG , Stice SS . Development and differentiation of neural rosettes derived from human embryonic stem cells. Stem Cell Rev. 2006;2:67‐77. 17142889 10.1007/s12015-006-0011-1

[68] Chambers SM , Fasano CA , Papapetrou EP , Tomishima M , Sadelain M , Studer L . Highly efficient neural conversion of human ES and iPS cells by dual inhibition of SMAD signaling. Nat Biotechnol. 2009;27:275‐280. 19252484 10.1038/nbt.1529 PMC2756723

[69] Elkabetz Y , Panagiotakos G , al Shamy G , Socci ND , Tabar V , Studer L . Human ES cell‐derived neural rosettes reveal a functionally distinct early neural stem cell stage. Genes Dev. 2008;22:152‐165. 18198334 10.1101/gad.1616208 PMC2192751

[70] Lancaster MA , Renner M , Martin CA , et al. Cerebral organoids model human brain development and microcephaly. Nature. 2013;501:373‐379. 23995685 10.1038/nature12517 PMC3817409

[71] Arlotta P , Paşca SP . Cell diversity in the human cerebral cortex: from the embryo to brain organoids. Curr Opin Neurobiol. 2019;56:194‐198. 31051421 10.1016/j.conb.2019.03.001

[72] Kelava I , Lancaster MA . Dishing out mini‐brains: current progress and future prospects in brain organoid research. Dev Biol. 2016;420:199‐209. 27402594 10.1016/j.ydbio.2016.06.037 PMC5161139

[73] Di Lullo E , Kriegstein AR . The use of brain organoids to investigate neural development and disease. Nat Rev Neurosci. 2017;18:573‐584. 28878372 10.1038/nrn.2017.107 PMC5667942

[74] Qian X , Nguyen HN , Song MM , et al. Brain‐region‐specific organoids using mini‐bioreactors for modeling ZIKV exposure. Cell. 2016;165:1238‐1254. 27118425 10.1016/j.cell.2016.04.032 PMC4900885

[75] Briscoe J , Ericson J . Specification of neuronal fates in the ventral neural tube. Curr Opin Neurobiol. 2001;11:43‐49. 11179871 10.1016/s0959-4388(00)00172-0

[76] Patten I , Placzek M . The role of sonic hedgehog in neural tube patterning. Cell Mol Life Sci. 2000;57:1695‐1708. 11130176 10.1007/PL00000652 PMC11146859

[77] Furuta Y , Piston DW , Hogan B . Bone morphogenetic proteins (BMPs) as regulators of dorsal forebrain development. Development. 1997;124:2203‐2212. 9187146 10.1242/dev.124.11.2203

[78] Mehler MF , Mabie PC , Zhang D , Kessler JA . Bone morphogenetic proteins in the nervous system. Trends Neurosci. 1997;20:309‐317. 9223224 10.1016/s0166-2236(96)01046-6

[79] Yoon S‐J , Elahi LS , Pașca AM , et al. Reliability of human cortical organoid generation. Nat Methods. 2019;16:75‐78. 30573846 10.1038/s41592-018-0255-0 PMC6677388

[80] Wang Y , Wang L , Zhu Y , Qin J . Human brain organoid‐on‐a‐chip to model prenatal nicotine exposure. Lab Chip. 2018;18:851‐860. 29437173 10.1039/c7lc01084b

[81] Kirkeby A , Grealish S , Wolf DA , et al. Generation of regionally specified neural progenitors and functional neurons from human embryonic stem cells under defined conditions. Cell Rep. 2012;1:703‐714. 22813745 10.1016/j.celrep.2012.04.009

[82] Muguruma K , Nishiyama A , Kawakami H , Hashimoto K , Sasai Y . Self‐organization of polarized cerebellar tissue in 3D culture of human pluripotent stem cells. Cell Rep. 2015;10:537‐550. 25640179 10.1016/j.celrep.2014.12.051

[83] Nayler S , Agarwal D , Curion F , Bowden R , Becker EB . High‐resolution transcriptional landscape of xeno‐free human induced pluripotent stem cell‐derived cerebellar organoids. Sci Rep. 2021;11:1‐17. 34155230 10.1038/s41598-021-91846-4 PMC8217544

[84] Xiang Y , Tanaka Y , Cakir B , et al. hESC‐derived thalamic organoids form reciprocal projections when fused with cortical organoids. Cell Stem Cell. 2019;24:487‐497.e487. 30799279 10.1016/j.stem.2018.12.015 PMC6853597

[85] Huang W‐K , Wong SZH , Pather SR , et al. Generation of hypothalamic arcuate organoids from human induced pluripotent stem cells. Cell Stem Cell. 2021;28:1657‐1670.e1610. 33961804 10.1016/j.stem.2021.04.006 PMC8419002

[86] Lancaster MA , Knoblich JA . Generation of cerebral organoids from human pluripotent stem cells. Nat Protoc. 2014;9:2329‐2340. 25188634 10.1038/nprot.2014.158 PMC4160653

[87] Quadrato G , Nguyen T , Macosko EZ , et al. Cell diversity and network dynamics in photosensitive human brain organoids. Nature. 2017;545:48‐53. 28445462 10.1038/nature22047 PMC5659341

[88] Camp JG , Badsha F , Florio M , et al. Human cerebral organoids recapitulate gene expression programs of fetal neocortex development. Proc Natl Acad Sci USA. 2015;112:15672‐15677. 26644564 10.1073/pnas.1520760112 PMC4697386

[89] Ormel PR , Vieira de Sá R , van Bodegraven EJ , et al. Microglia innately develop within cerebral organoids. Nat Commun. 2018;9:1‐14. 30301888 10.1038/s41467-018-06684-2 PMC6177485

[90] Mansour AA , Schafer ST , Gage, FH . Cellular complexity in brain organoids: current progress and unsolved issues. Semin Cell Dev Biol. 2021;111:32‐39. 32499191 10.1016/j.semcdb.2020.05.013

[91] Bodnar B , Zhang Y , Liu J , et al. Novel scalable and simplified system to generate microglia‐containing cerebral organoids from human induced pluripotent stem cells. Front Cell Neurosci. 2021;206:682272. 10.3389/fncel.2021.682272 PMC8288463 34290591

[92] Gopalakrishnan J . The emergence of stem cell‐based brain organoids: trends and challenges. Bioessays. 2019;41:1900011. 10.1002/bies.201900011 31274205

[93] Xiang Y , Cakir B , Park I‐H . Deconstructing and reconstructing the human brain with regionally specified brain organoids. Semin Cell Dev Biol. 2021;111:40‐51. 32553582 10.1016/j.semcdb.2020.05.023 PMC12581431

[94] Jacob F , Schnoll JG , Song H , Ming G‐l . Building the brain from scratch: engineering region‐specific brain organoids from human stem cells to study neural development and disease. Curr Top Dev Biol. 2021;142:477‐530. 33706925 10.1016/bs.ctdb.2020.12.011 PMC8363060

[95] Son MY , Sim H , Son YS , et al. Distinctive genomic signature of neural and intestinal organoids from familial Parkinson's disease patient‐derived induced pluripotent stem cells. Neuropathol Appl Neurobiol. 2017;43:584‐603. 28235153 10.1111/nan.12396

[96] Kim H , Park HJ , Choi H , et al. Modeling G2019S‐LRRK2 sporadic Parkinson's disease in 3D midbrain organoids. Stem Cell Rep. 2019;12:518‐531. 10.1016/j.stemcr.2019.01.020 PMC6410341 30799274

[97] Conforti P , Besusso D , Bocchi VD , et al. Faulty neuronal determination and cell polarization are reverted by modulating HD early phenotypes. Proc Natl Acad Sci USA. 2018;115:E762‐E771. 29311338 10.1073/pnas.1715865115 PMC5789931

[98] Zhang J , Ooi J , Utami KH , et al. Expanded huntingtin CAG repeats disrupt the balance between neural progenitor expansion and differentiation in human cerebral organoids. bioRxiv. 2019;850586.

[99] Groveman BR , Foliaki ST , Orru CD , et al. Sporadic Creutzfeldt‐Jakob disease prion infection of human cerebral organoids. Acta Neuropathol Commun. 2019;7:1‐12. 10.1186/s40478-019-0742-2 PMC6567389 31196223

[100] Zhang L , Chen C , Mak MSH , et al. Advance of sporadic Alzheimer's disease animal models. Med Res Rev. 2020;40:431‐458. 31328804 10.1002/med.21624

[101] Raja WK , Mungenast AE , Lin YT , et al. Self‐organizing 3D human neural tissue derived from induced pluripotent stem cells recapitulate Alzheimer's disease phenotypes. PLoS One. 2016;11:e0161969. 27622770 10.1371/journal.pone.0161969 PMC5021368

[102] Gonzalez C , Armijo E , Bravo‐Alegria J , Becerra‐Calixto A , Mays CE , Soto C . Modeling amyloid beta and tau pathology in human cerebral organoids. Mol Psychiatry. 2018;23:2363‐2374. 30171212 10.1038/s41380-018-0229-8 PMC6594704

[103] Yan Y , Song L , Bejoy J , et al. Modeling neurodegenerative microenvironment using cortical organoids derived from human stem cells. Tissue Eng Part A. 2018;24:1125‐1137. 29361890 10.1089/ten.tea.2017.0423 PMC6033307

[104] Ghatak S , Dolatabadi N , Trudler D , et al. Mechanisms of hyperexcitability in Alzheimer's disease hiPSC‐derived neurons and cerebral organoids vs isogenic controls. Elife. 2019;8:e50333. 31782729 10.7554/eLife.50333 PMC6905854

[105] Hernández‐Sapiéns MA , Reza‐Zaldívar EE , Cevallos RR , Márquez‐Aguirre AL , Gazarian K , Canales‐Aguirre AA . A three‐dimensional Alzheimer's disease cell culture model using iPSC‐derived neurons carrying A246E mutation in PSEN1. Front Cell Neurosci. 2020;14:151. 32655369 10.3389/fncel.2020.00151 PMC7325960

[106] Kuehner JN , Chen J , Bruggeman EC , et al. 5‐hydroxymethylcytosine is dynamically regulated during forebrain organoid development and aberrantly altered in Alzheimer's disease. Cell Rep. 2021;35:109042. 33910000 10.1016/j.celrep.2021.109042 PMC8106871

[107] Yin J , VanDongen AM . Enhanced neuronal activity and asynchronous calcium transients revealed in a 3D organoid model of Alzheimer's disease. ACS Biomater Sci Eng. 2020;7:254‐264. 33347288 10.1021/acsbiomaterials.0c01583

[108] Bloom GS . Amyloid‐β and tau: the trigger and bullet in Alzheimer disease pathogenesis. JAMA Neurol. 2014;71:505‐508. 24493463 10.1001/jamaneurol.2013.5847 PMC12908160

[109] Martineau M , Guzman RE , Fahlke C , Klingauf J . VGLUT1 functions as a glutamate/proton exchanger with chloride channel activity in hippocampal glutamatergic synapses. Nat Commun. 2017;8:1‐13. 29273736 10.1038/s41467-017-02367-6 PMC5741633

[110] Sokolow S , Luu SH , Nandy K , et al. Preferential accumulation of amyloid‐beta in presynaptic glutamatergic terminals (VGluT1 and VGluT2) in Alzheimer's disease cortex. Neurobiol Dis. 2012;45:381‐387. 21914482 10.1016/j.nbd.2011.08.027 PMC3339276

[111] Timmer NM , Metaxas A , van der Stelt I , Kluijtmans LAJ , van Berckel BN , Verbeek MM . Cerebral level of vGlut1 is increased and level of glycine is decreased in TgSwDI mice. J Alzheimers Dis. 2014;39:89‐101. 24145381 10.3233/JAD-130437

[112] Popugaeva E , Pchitskaya E , Bezprozvanny I . Dysregulation of intracellular calcium signaling in Alzheimer's disease. Antioxid Redox Signal. 2018;29:1176‐1188. 29890840 10.1089/ars.2018.7506 PMC6157344

[113] Lovejoy CEJ . iPSC Derived Cerebral Organoids as a Model of Tauopathy. UCL (University College London); 2021.

[114] Lin Y‐T , Seo J , Gao F , et al. APOE4 causes widespread molecular and cellular alterations associated with Alzheimer's disease phenotypes in human iPSC‐derived brain cell types. Neuron. 2018;98:1141‐1154.e1147. 29861287 10.1016/j.neuron.2018.05.008 PMC6023751

[115] Hernández D , Rooney LA , Daniszewski M , et al. Culture variabilities of human iPSC‐derived cerebral organoids are a major issue for the modelling of phenotypes observed in Alzheimer's disease. Stem Cell Rev Rep. 2021;18:718‐731. 33725267 10.1007/s12015-021-10147-5

[116] Zhao J , Fu Y , Yamazaki Y , et al. APOE4 exacerbates synapse loss and neurodegeneration in Alzheimer's disease patient iPSC‐derived cerebral organoids. Nat Commun. 2020;11:1‐14. 33139712 10.1038/s41467-020-19264-0 PMC7608683

[117] Park J‐C , Jang SY , Lee D , et al. A logical network‐based drug‐screening platform for Alzheimer's disease representing pathological features of human brain organoids. Nat Commun. 2021;12:1‐13. 33436582 10.1038/s41467-020-20440-5 PMC7804132

[118] Blanchard JW , Bula M , Davila‐Velderrain J , et al. Reconstruction of the human blood–brain barrier in vitro reveals a pathogenic mechanism of APOE4 in pericytes. Nat Med. 2020;26:952‐963. 32514169 10.1038/s41591-020-0886-4 PMC7704032

[119] Smith EE , Greenberg SM . β‐Amyloid, blood vessels, and brain function. Stroke. 2009;40:2601‐2606. 19443808 10.1161/STROKEAHA.108.536839 PMC2704252

[120] Chen X , Sun G , Tian E , et al. Modeling sporadic Alzheimer's disease in human brain organoids under serum exposure. Adv Sci. 2021;8:2101462. 10.1002/advs.202101462 PMC8456220 34337898

[121] Pavoni S , Jarray R , Nassor F , et al. Small‐molecule induction of Aβ‐42 peptide production in human cerebral organoids to model Alzheimer's disease associated phenotypes. PLoS One. 2018;13:e0209150. 30557391 10.1371/journal.pone.0209150 PMC6296660

[122] Bejoy J , Song L , Wang Z , Sang QX , Zhou Y , Li Y . Neuroprotective activities of heparin, heparinase III, and hyaluronic acid on the Aβ42‐treated forebrain spheroids derived from human stem cells. ACS Biomater Sci Eng. 2018;4:2922‐2933. 30533518 10.1021/acsbiomaterials.8b00021 PMC6286050

[123] Cairns DM , Rouleau N , Parker RN , Walsh KG , Gehrke L , Kaplan DL . A 3D human brain‐like tissue model of herpes‐induced Alzheimer's disease. Sci Adv. 2020;6:eaay8828. 32494701 10.1126/sciadv.aay8828 PMC7202879

[124] Pérez MJ , Ivanyuk D , Panagiotakopoulou V , et al. Loss of function of the mitochondrial peptidase PITRM1 induces proteotoxic stress and Alzheimer's disease‐like pathology in human cerebral organoids. Mol Psychiatry. 2020;1‐18:5733‐5750. 10.1038/s41380-020-0807-4 PMC8758476 32632204

[125] Hochard A , Oumata N , Bettayeb K , et al. Aftins increase amyloid‐β 42, lower amyloid‐β 38, and do not Alter amyloid‐β 40 extracellular production in vitro: toward a chemical model of Alzheimer's disease? J Alzheimers Dis. 2013;35:107‐120. 23364140 10.3233/JAD-121777 PMC5039020

[126] Itzhaki RF . Corroboration of a major role for herpes simplex virus type 1 in Alzheimer's disease. Front Aging Neurosci. 2018;10:324. 30405395 10.3389/fnagi.2018.00324 PMC6202583

[127] Qiao H , Zhao W , Guo M , et al. Cerebral organoids for modeling of HSV‐1‐induced‐multiscale neuropathology associated with Alzheimer's disease and phenotypic rescue. bioRxiv. 2022. 10.3390/ijms23115981 PMC9181143 35682661

[128] Chen M , Lee HK , Moo L , Hanlon E , Stein T , Xia W . Common proteomic profiles of induced pluripotent stem cell‐derived three‐dimensional neurons and brain tissue from Alzheimer patients. J Proteomics. 2018;182:21‐33. 29709615 10.1016/j.jprot.2018.04.032 PMC7457321

[129] Alić I , Goh PA , Murray A , et al. Patient‐specific Alzheimer‐like pathology in trisomy 21 cerebral organoids reveals BACE2 as a gene dose‐sensitive AD suppressor in human brain. Mol Psychiatry. 2020;26:5766‐5788. 32647257 10.1038/s41380-020-0806-5 PMC8190957

[130] Cai H , Ao Z , Hu L , et al. Acoustofluidic assembly of 3D neurospheroids to model Alzheimer's disease. Analyst. 2020;145:6243‐6253. 32840509 10.1039/d0an01373k PMC7530134

[131] Mansour AA , Gonçalves JT , Bloyd CW , et al. An in vivo model of functional and vascularized human brain organoids. Nat Biotechnol. 2018;36:432‐441. 29658944 10.1038/nbt.4127 PMC6331203

[132] Shin N , Kim Y , Ko J , et al. Vascularization of iNSC spheroid in a 3D spheroid‐on‐a‐chip platform enhances neural maturation. Biotechnol Bioeng. 2022;119:566‐574. 34716703 10.1002/bit.27978 PMC9298365

[133] Xu R , Boreland AJ , Li X , et al. Developing human pluripotent stem cell‐based cerebral organoids with a controllable microglia ratio for modeling brain development and pathology. Stem Cell Rep. 2021;16:1923‐1937. doi:10.1016/j.stemcr.2021.06.011 PMC8365109 34297942

[134] Song L , Yuan X , Jones Z , et al. Functionalization of brain region‐specific spheroids with isogenic microglia‐like cells. Sci Rep. 2019;9:1‐18. 31363137 10.1038/s41598-019-47444-6 PMC6667451

[135] Nzou G , Wicks RT , Wicks EE , et al. Human cortex spheroid with a functional blood brain barrier for high‐throughput neurotoxicity screening and disease modeling. Sci Rep. 2018;8:1‐10. 29743549 10.1038/s41598-018-25603-5 PMC5943588

[136] Cho A‐N , Jin Y , An Y , et al. Microfluidic device with brain extracellular matrix promotes structural and functional maturation of human brain organoids. Nat Commun. 2021;12:1‐23. 34354063 10.1038/s41467-021-24775-5 PMC8342542

[137] Chiaradia I , Lancaster MA . Brain organoids for the study of human neurobiology at the interface of in vitro and in vivo. Nat Neurosci. 2020;23:1496‐1508. doi:10.1038/s41593-020-00730-3 33139941

[138] Aisenbrey EA , Murphy WL . Synthetic alternatives to Matrigel. Nat Rev Mater. 2020;5:539‐551. doi:10.1038/s41578-020-0199-8 32953138 PMC7500703

[139] Cassel de Camps C , Aslani S , Stylianesis N , et al. Hydrogel mechanics influence the growth and development of embedded brain organoids. ACS Appl Bio Mater. 2022;5:214‐224. doi:10.1021/acsabm.1c01047 35014820

[140] Raimondi I , Tunesi M , Forloni G , Albani D , Giordano C . 3D brain tissue physiological model with co‐cultured primary neurons and glial cells in hydrogels. J Tissue Eng. 2020;11:2041731420963981. doi:10.1177/2041731420963981 33117519 PMC7570768

[141] Ranga A , Girgin M , Meinhardt A , et al. Neural tube morphogenesis in synthetic 3D microenvironments. Proc Natl Acad Sci USA. 2016;113:E6831‐E6839. doi:10.1073/pnas.1603529113 27742791 PMC5098636

[142] Lindborg BA , Brekke JH , Vegoe AL , et al. Rapid induction of cerebral organoids from human induced pluripotent stem cells using a chemically defined hydrogel and defined cell culture medium. Stem Cells Transl Med. 2016;5:970‐979. doi:10.5966/sctm.2015-0305 27177577 PMC4922855

[143] Oksdath M , Perrin SL , Bardy C , et al. Review: Synthetic scaffolds to control the biochemical, mechanical, and geometrical environment of stem cell‐derived brain organoids. APL Bioeng. 2018;2:041501. doi:10.1063/1.5045124 31069322 PMC6481728

[144] Simsa R , Rothenbücher T , Gürbüz H , et al. Brain organoid formation on decellularized porcine brain ECM hydrogels. PLoS One. 2021;16:e0245685. doi:10.1371/journal.pone.0245685 33507989 PMC7842896

[145] Blander JM , Longman RS , Iliev ID , Sonnenberg GF , Artis D . Regulation of inflammation by microbiota interactions with the host. Nat Immunol. 2017;18:851‐860. 28722709 10.1038/ni.3780 PMC5800875

[146] Burke R , Gillies G , Hales S , Sullivan A , Tofler G , Gallagher R . Cognitive impairment in heart failure patients. Heart Lung Circ. 2012;21:S76.

[147] Cattaneo A , Cattane N , Galluzzi S , et al. Association of brain amyloidosis with pro‐inflammatory gut bacterial taxa and peripheral inflammation markers in cognitively impaired elderly. Neurobiol Aging. 2017;49:60‐68. 27776263 10.1016/j.neurobiolaging.2016.08.019

[148] De Deyn PP , Vanholder R , Eloot S , Glorieux G . Progress in uremic toxin research: guanidino compounds as uremic (neuro) toxins. Seminars in Dialysis. Vol 22. Blackwell Publishing Ltd; 2009:340‐345. 19708978 10.1111/j.1525-139X.2009.00577.x

[149] Deckers K , Camerino I , van Boxtel MPJ , et al. Dementia risk in renal dysfunction: a systematic review and meta‐analysis of prospective studies. Neurology. 2017;88:198‐208. 27974647 10.1212/WNL.0000000000003482 PMC5224710

[150] Lovell J , Pham T , Noaman SQ , Davis MC , Johnson M , Ibrahim JE . Self‐management of heart failure in dementia and cognitive impairment: a systematic review. BMC Cardiovasc Disord. 2019;19:1‐18. 31035921 10.1186/s12872-019-1077-4 PMC6489234

[151] Minter MR , Zhang C , Leone V , et al. Antibiotic‐induced perturbations in gut microbial diversity influences neuro‐inflammation and amyloidosis in a murine model of Alzheimer's disease. Sci Rep. 2016;6:1‐12. 27443609 10.1038/srep30028 PMC4956742

[152] Zhan X , Stamova B , Jin LW , DeCarli C , Phinney B , Sharp FR . Gram‐negative bacterial molecules associate with Alzheimer disease pathology. Neurology. 2016;87:2324‐2332. 27784770 10.1212/WNL.0000000000003391 PMC5135029

[153] Calvani R , Picca A , Lo Monaco MR , Landi F , Bernabei R , Marzetti E . Of microbes and minds: a narrative review on the second brain aging. Front Med. 2018;5:53. 10.3389/fmed.2018.00053 PMC5840854 29552561

[154] Bi F‐C , Yang XH , Cheng XY , et al. Optimization of cerebral organoids: a more qualified model for Alzheimer's disease research. Transl Neurodegen. 2021;10:1‐13. 10.1186/s40035-021-00252-3 PMC8349709 34372927

